# Nonlinear connectedness of conventional crypto-assets and sustainable crypto-assets with climate change: A complex systems modelling approach

**DOI:** 10.1371/journal.pone.0318647

**Published:** 2025-02-07

**Authors:** Mushtaq Hussain Khan, Shreya Macherla, Angesh Anupam

**Affiliations:** 1 Cardiff School of Management, Cardiff Metropolitan University, Cardiff, United Kingdom; 2 Cardiff School of Technologies, Cardiff Metropolitan University, Cardiff, United Kingdom; IIM Sambalpur, INDIA

## Abstract

Earlier studies used classical time series models to forecast the nonlinear connectedness of conventional crypto-assets with CO_2_ emissions. For the first time, this study aims to provide a data-driven Nonlinear System Identification technique to study the nonlinear connectedness of crypto-assets with CO_2_ emissions. Using daily data from January 2, 2019, to March 31, 2023, we investigate the nonlinear connectedness among conventional crypto-assets, sustainable crypto-assets, and CO_2_ emissions based on our proposed model, Multiple Inputs Single Output (MISO) Nonlinear Autoregressive with Exogenous Inputs (NARX). Intriguingly, the forecasting accuracy of the proposed model improves with the inclusion of exogenous input variables (conventional and sustainable crypto-assets). Overall, our results reveal that conventional crypto-assets exhibit slightly stronger connectedness with CO_2_ emissions compared to sustainable crypto-assets. These findings suggest that, to some extent, sustainable crypto-assets provide a solution to the environmental issues related to CO_2_ emissions. However, further improvements in sustainable crypto-assets through technological advances are required to develop more energy-efficient decentralised finance consensus algorithms, with the aim of reshaping the cryptocurrency ecosystem into an environmentally sustainable market.

## Introduction

In the context of global climate change, imminent action is necessary for the reduction of carbon emissions in accordance with the Paris Agreement (12 December 2015), especially with regard to the attainment of the Sustainable Development Goals (SDGs). In recent decades, cryptocurrency mining has been considered a major source of carbon emissions because rapid growth in the cryptocurrency market leads to a sharp rise in computing power requirements for handling mining machines and the amount of energy required to generate digital tokens [1, 2]. According to the findings of the Intergovernmental Panel on Climate Change (IPCC, 2018), a rise in global temperatures by 1.5°C compared to pre-industrial levels, primarily driven by a significant surge in global CO_2_ emissions, could result in irreversible environmental impacts. This could encompass the potential loss of Arctic ice and a rise in sea levels. Moreover, urgent and unified efforts are crucial to swiftly transition towards carbon neutrality, aiming to mitigate the negative effects of CO_2_ emissions and tackle sustainability concerns, as underscored by the IPCC in 2022. In this context, the Bitcoin Electricity Consumption Index (https://ccaf.io/cbnsi/cbeci) by Cambridge University revealed that approximately 0.38% of the world’s electricity is utilised for Bitcoin mining. This surpasses the electricity consumption of entire nations such as Belgium and Finland.

Contrary to conventional cryptocurrencies, recent developments in the cryptocurrency market following the Crypto Climate Accord led by the private sector aimed at decarbonisation of the global crypto and blockchain sector by 2030. The cryptocurrencies follow the Crypto Climate Accord, we call these cryptocurrencies as sustainable crypto-assets. The major examples of sustainable crypto-assets are green cryptocurrencies, renewable energy tokens, and Islamic Defi tokens, among others. Empirical studies have investigated the environmental impacts of cryptocurrencies, for instance, the sustainability of Bitcoin and blockchains [3], Bitcoin emissions and global warming [4, 5], carbon footprint emissions from Bitcoin mining [6, 7], Bitcoin mining and environmental concerns [8], asymmetric effects of crypto-assets on environmental sustainability [9], cryptocurrency return predictability and CO_2_ emissions [10], water and carbon footprint of cryptocurrencies [11], cryptocurrency mining, power market stability and climate perspectives [12], cryptocurrencies and global sustainability [13], and Bitcoin mining and climate change [14].

Motivated by Zhang et al. [14] and in light of the growing concerns regarding the environmental impacts of cryptocurrencies, this study explores whether the connection between conventional crypto-assets and CO_2_ emissions differs from that of sustainable crypto-assets. Our study extends the important work of Zhang et al. [14] in the following ways. First, they considered only the conventional crypto-asset, Bitcoin. However, our study compares the environmental impacts of conventional crypto-assets with those of sustainable crypto-assets. Although sustainable crypto-assets align with the Crypto Climate Accord, they still face several limitations and technological hurdles, particularly in their efforts to reduce CO_2_ emissions. One major challenge is the energy-intensive nature of blockchain technologies, especially those relying on proof-of-work (PoW) consensus mechanisms [29]. They further argue that mining cryptocurrencies consumes vast amounts of electricity, much of which is still derived from non-renewable energy sources. Transitioning to more sustainable alternatives, such as proof-of-stake (PoS) or other energy-efficient consensus mechanisms, requires significant technological innovation and widespread adoption, which can be slow and contentious within decentralised communities.

Additionally, implementing renewable energy solutions for crypto mining operations poses logistical and financial barriers, as renewable infrastructure often demands substantial upfront investment and may not be equally accessible across regions. Furthermore, tracking and verifying the carbon footprint of crypto-assets in real-time necessitates advanced monitoring systems and enhanced transparency. These limitations underscore the need for coordinated global efforts, policy reforms, and technological advancements to align crypto-assets with sustainable development goals and mitigate the environmental impact of cryptocurrencies.

Second, they utilised daily world CO_2_ emissions data from the residential sector, while our study links daily trading activity for both conventional and sustainable crypto-assets with daily world CO_2_ emissions from the power sector. We argue that CO_2_ emissions from the power sector may provide a more accurate representation of the environmental impact of cryptocurrencies, as it accounts for the energy generated from the power requirements of mining machines to generate digital tokens. Third, Zhang et al. [14] used the classical Granger causality tests and the Vector autoregressive (VAR) model to investigate nonlinear connections between Bitcoin and CO_2_ emissions. In contrast, we employ a more advanced and robust approach based on Nonlinear Systems Identification, Complex Systems Modelling (Multiple Inputs Single Output (MISO) Nonlinear Autoregressive Network with Exogenous Inputs (NARX) model, to explore the connectedness between crypto-assets and CO_2_ emissions. The NARX models comprising of nonlinear regressors are employed as part of the Nonlinear Systems Identification methodology. This model can explain the nonlinear dynamical relationship among the independent and dependent variables whilst revealing the contribution of each term on an incremental basis. The transparency in the NARX model structure serves as a tool for optimising the independent variables for a more desired level of the output of interest. It is noteworthy to mention that the NARX model structure selection algorithm adopted in this study is capable of generating a linear ARX model if the linear terms are adequate to explain the connection between power emission and crypto trading. This approach does not rule out the possibility of a linear relationship among the exogenous input variables [15].

Several variables show leptokurtic behaviour with a fat tail and sharp peakedness (non-normally distributed data). Thus, we consider the Nonlinear System Identification technique, MISO–NARX model in this study to address the connectedness of conventional and sustainable crypto-assets with CO_2_ emissions. Based on the MISO–NARX model, we find that the connectedness of conventional crypto-assets with CO_2_ emissions is slightly stronger compared to sustainable crypto-assets. Interestingly, the inclusion of both conventional and sustainable crypto-assets as exogenous input variables enhances the goodness of fit for our suggested model. Alternatively, the variance in CO_2_ emissions is greatly explained whilst all the input variables are considered.

The contribution of this study is twofold. First, this study extends the emerging literature on cryptocurrencies and CO_2_ emissions nexus. Specifically, we complement the works of [5–10, 14], among others. Second, this study offers a methodological contribution by employing a data-driven approach to analyse the environmental impacts of both conventional and sustainable crypto-assets. This approach helps to minimise or eliminate errors caused by model misspecifications and the use of inconsistent estimators [16, 17]. Data-driven approaches have been used in the context of conventional cryptocurrencies and carbon emissions [18–21]; however, there is a lack of evidence on the use of such approaches to explore the nonlinear dynamics of the sustainable cryptocurrencies and carbon emissions nexus. Therefore, a dynamical model is employed using the system identification methodology by deriving a NARX model structure. The Forward Regression Orthogonal Least Square (FROLS) algorithm is employed for selecting the optimal model terms, which is implemented using SysIdentPy, a Python library.

The rest of the study is organised as follows. Section 2 provides the recent literature on the environmental impacts of cryptocurrencies. Section 3 describes the data and methodology. Section 4 discusses the empirical results and section 5 provides concluding remarks.

## Literature review and hypotheses development

The implications of financial technology (crypto-assets) on the environment is an emerging topic among academicians and policymakers. This is because the advancements in technology, particularly in the realm of digital finance, offer novel opportunities but are accompanied by environmental repercussions. The cryptocurrency ecosystem has experienced a significant surge in market capitalisation over the past decade. The negative environmental effects are extensive and can result in increased occurrences of extreme weather events, presenting notable threats to public health [14]. The rapid expansion of both blockchain technology and the cryptocurrency market presents a significant challenge to global initiatives aimed at mitigating CO_2_ emissions, as noted by Jiang [22] and Truby [23]. Hence, it is crucial to understand the environmental impacts of advancements in digital finance within the framework of climate change.

Numerous studies have shed light on the link between cryptocurrency energy consumption particularly Bitcoin’s carbon footprint and the environment [6, 14, 24–27], and the findings of these studies are mixed. The seminal work of O’Dwyer and Malone [28] has started the important emerging debate regarding the energy and environmental footprint of cryptocurrencies. They noted that the power consumption for Bitcoin mining is equivalent to the electricity consumption of Ireland. In the context of this research, De Vries et al. [7] also reported that the yearly carbon emissions and electricity consumption associated with Bitcoin amount to 76.67 Mt of CO_2_ and 132.07 TWh, which is equivalent to the carbon emissions of Turkmenistan and the electricity consumption of Argentina, respectively.

Wendl et al. [29] conducted a systematic literature review on the environmental impact of cryptocurrencies using proof of stake (PoS) and proof of work (PoW) algorithms. They noted that PoW cryptocurrencies, particularly Bitcoin, are associated with an ever-increasing environmental impact, while PoS cryptocurrencies serve as sustainable alternatives. Likewise, Gallersdörfer et al. [30] observed that among the top 20 cryptocurrencies, Bitcoin accounts for two-thirds of the total energy consumption in the cryptocurrency space. Malfuzi et al. [31] observed that in October 2019, Bitcoin mining consumed significantly more energy than the energy consumption of Austria. Similarly, Badea and Mungiu-Pupazan [32] reported that each Bitcoin transaction contributes to approximately 619Kwt of carbon emissions. This is equivalent to the environmental footprint generated by 350,000 bank card transactions or the energy consumption of an average USA family over a period of 20.92 days. In the case of Ethereum, in June 2017, the entire Ethereum network consumed an amount of electricity comparable to that of a relatively small country, like Cyprus, as pointed out by Corbet and Yarovaya [33].

Empirically, Zhang et al. [14] employed the Granger causality tests and the Vector autoregressive (VAR) model to examine the dynamic connectedness between Bitcoin mining and climate change. They found significant Granger causality between climate change and the energy usage of Bitcoin mining. In the case of dynamic connectedness, they noted that the hash rate transmits the most substantial net spillover effects to Bitcoin electricity consumption and climate change. Likewise, Lorente et al. [34] utilised the Cambridge Bitcoin Consumption Index to investigate the relationship between cryptocurrency energy consumption and its impact on climate change, clean energy, and green financial markets. By employing the Quantile Vector Autoregressive approach, they observed that the Bitcoin energy consumption index appears to be a net contributor to shocks at both lower and upper quantiles for other variables.

Kamal and Hassan [35] used the Cryptocurrency Environmental Attention Index (ICEA) introduced by Wang et al. [36], to examine the asymmetric connectedness of green assets with ICEA. They used a quantile connectedness approach and dynamic conditional correlations (DCC)-GJR-GARCH model and found a significant dynamic connectedness of green assets with ICEA. They found significant dynamic connectedness between green assets and ICEA by employing a quantile connectedness approach and dynamic conditional correlations (DCC)-GJR-GARCH model. Husain et al. [37] used wavelet coherence analysis to test the dynamic connectedness of green cryptocurrencies, green investment, conventional commodities and equities over the period of 2018 to 2023. In contrast to earlier studies, they noted that green cryptocurrencies do not display hedging or safe haven properties; rather, they behave no differently than diversifiers. Mustafa et al. [38], through a systematic literature review and bibliometric analysis, identified the potential linkage between cryptocurrency trading and UN Sustainable Development Goals, specifically SDG 7 (affordable and clean energy) and SDG 13 (climate action). Alzoubi and Mishra [39] also conducted a systematic literature review to identify blockchain networks and systems that claimed to be environmentally friendly. They identified 23 blockchain networks that consume substantially less energy and emit fewer carbon dioxide emissions in comparison to the Bitcoin network. Erdogan et al. [9] investigated the connection between cryptocurrencies and environmental degradation by employing standard and asymmetric causality methods, the Toda-Yamamoto, bootstrap-augmented Toda-Yamamoto and Fourier-augmented Toda-Yamamoto. Their results revealed that Bitcoin and Ethereum have significant causal effects on environmental degradation. In their study, Marco et al. [40] employed a time-frequency quantile connectedness approach to investigate the return connectedness between cryptocurrencies and the U.S. environmental stock market indexes. They found significant linkages between cryptocurrencies and the U.S. environmental stock market indexes, both in the short and long run.

It is noteworthy that none of the above-mentioned studies shed light on the differential impact of conventional crypto-assets and sustainable crypto-assets on the environment in nonlinear settings. This study, therefore, aims to address such gaps in the literature. Hence, this is the first empirical study accounting for the nonlinear connectedness among conventional crypto-assets, sustainable crypto-assets, and CO_2_ emissions using robust complex systems modelling, MISO–NARX. Importantly, we posit a weak connection between sustainable crypto-assets and CO_2_ emissions. Alternatively, sustainable crypto-assets may mitigate environmental concerns within the cryptocurrency ecosystem through sustainability measures. Based on the above discussion, we formulate our hypotheses as follows:

***Hypothesis 1a*:** *There exists a connectedness between crypto-assets and CO*_*2*_ *emissions*.
***Hypothesis 1b*:** *The effect of sustainable crypto-assets on CO*_*2*_ *emissions is significantly different than conventional crypto-assets*.

## Data and methodology

### Construction of the sample and data sources

This study attempts to unravel the nonlinear connectedness of conventional and sustainable cryptocurrencies with CO_2_ emissions. We follow Zhang et al. [14] and use the daily world CO_2_ emissions. Crypto-trading is measured by the trading activity/volume of crypto assets. Specifically, five conventional crypto-assets namely Binance (BNB-USD), Bitcoin BTC-USD, Ethereum (ETH-USD), Tether (USDT-USD), and Ripple (XRP-USD) and five sustainable crypto-assets namely Cardano (ADA-USD), Bitcoin Green (BITG-USD), IOTA (MIOTA-USD), Nano (XNO-USD), and Powerledger (POWR-USD) were considered for empirical analysis. The data regarding CO_2_ emissions are obtained from the website of Carbon Monitor (https://carbonmonitor.org/), while the trading volume data of cryptocurrencies are obtained from the website of Coinmarket cap.com (https://coinmarketcap.com/) and Yahoo Finance. Moreover, world CO_2_ emission (per metric ton) for power sectors is used as a dependent variable, and crypto-assets trading volumes in U.S. dollar-term as an independent variable. By following Mohsin et al. [41] and Ante et al. [42] among others, all variables are converted into a natural logarithm to address skewness and kurtosis in the data [S1 Data]. Specifically, all variables are collected in raw format (daily volumes) and then converted to return series using the formula below.

$$R_{t}={lnP}_{t}-{lnP}_{t-1}$$

where is *R*_*t*_ return series, *lnP*_*t*_ is the natural log of the current volume of each series, whereas *lnP*_*t*−1_ is the natural log of the lagged volume of each series.

Table 1 presents the summary statistics of the variables used in the analysis. The sample period of the study ranges from January 2, 2019, to March 31, 2023, the number of observations for each variable is 1,511, and the total number of observations is 16,621. We can see that almost all crypto-assets exhibit variation, the mean values ranging from -0.000042 (ETH_USD) to 0.000767 (USDT_USD). Besides, the standard deviation ranged from 0.213776 (USDT_USD) to 1.023988 (POWR_USD) with minimum and maximum values of -7.577718 (POWR_USD) and 6.492619 (BITG_USD), respectively. In the case of the dependent variable (CO_2_ emissions), the standard deviation is 0.033142 with minimum and maximum values of -0.131578 and 0.113131, respectively.

**Table 1:** Summary statistics.

	Mean	Maximum	Minimum	Std. Dev.	Skewness	Kurtosis	Jarque-Bera	Prob.	Obs.
BNB_USD	0.000746	1.090492	-0.912855	0.255515	0.453946	4.334813	164.07	0.00	1511
BTC_USD	0.000456	1.862374	-2.033991	0.246745	-0.048269	9.202891	2422.96	0.00	1511
ETH_USD	-0.000042	1.012270	-0.827715	0.240339	0.196411	4.252219	108.44	0.00	1511
USDT_USD	0.000767	0.825359	-0.720289	0.213776	0.226085	3.858299	59.25	0.00	1511
XRP_USD	-0.000277	1.517027	-1.962618	0.320485	0.459457	5.761309	533.21	0.00	1511
ADA_USD	0.002234	1.154425	-1.134560	0.326868	0.410405	3.469882	56.32	0.00	1511
BITG_USD	-0.002331	6.492619	-7.222502	0.846357	0.255407	14.301800	8058.13	0.00	1511
MIOTA_USD	0.000009	2.774113	-1.730723	0.406835	0.668025	5.874800	632.70	0.00	1511
XNO_USD	-0.000869	4.875773	-1.947404	0.502140	1.429281	12.827860	6595.40	0.00	1511
POWR_USD	0.005471	6.291877	-7.577718	1.023988	0.395810	10.911690	3980.33	0.00	1511
CO_2_ emissions	-0.000003	0.113131	-0.131578	0.033142	0.394910	3.040445	39.38	0.00	1511

Most of the values are positively skewed and the kurtosis values show that the data follows leptokurtic behaviour (greater than 3) with a peaked, fat-tailed distribution. In addition, S1–S3 Figs exhibit volatility spikes in CO_2_ emissions, trading volumes of conventional and sustainable crypto-assets. Hence, simple regression-based estimation may produce incorrect results for non-normally distributed data. Therefore, the Nonlinear System Identification approach, MISO–NARX is used.

### Methodology

#### Nonlinear system identification

Nonlinear System Identification is a data-driven technique for the identification of a dynamical model corresponding to a nonlinear complex system. This method has its origin in Control Theory but has been applied in a wide range of disciplines [43] in the last few decades. Some of the examples are environmental systems [44], mechanical systems [45], biological systems [46–48], financial systems and energy [49–52], among others. The most common model class within Nonlinear System Identification is known as Nonlinear Autoregressive Moving Average with Exogenous Input (NARMAX) Model. The model structure of a typical NARMAX representation allows the capturing of complex nonlinear dynamics of the systems making it a reasonable option in modelling complex systems’ behaviour. In simple words, a typical NARMAX model is a nonlinear function of time-lagged exogenous inputs and output variables.

Although NARMAX is mainly employed for modelling the dynamics of nonlinear systems, if a system’s dynamics can be explained solely through linear terms, then NARMAX can be reduced to its linear variant, known as, ARMAX. The NARMAX modelling falls under the data-driven modelling paradigm and therefore the model accuracy is highly dependent upon the quality of data available during training of the model. Nonetheless, unlike a typical black box modelling approach, a differential equation or difference equation-based NARMAX allows a more transparent view of the underlying systems. This feature makes it possible to tease apart the characteristics of the system for potential optimisation along with forecasting the outputs of interest.

The NARMAX model is one of the most popular frameworks in a Nonlinear System Identification process. Its structure is uniquely suitable for capturing the complex dynamics of various kinds of real-world systems, making it an optimal choice for modelling and understanding complex system behaviours. A NARMAX model typically represents a nonlinear function that draws a relation between the system’s output to exogenous inputs and output variables. This helps in obtaining a robust representation of the system.

The use case of the NARMAX framework extends beyond predictive analytics. Whilst the majority of the machine learning-based modelling approaches focus on accurate prediction of future states, NARMAX models prioritise on understanding the underlying dynamics of the system. This characteristic makes them particularly suitable in use cases where drawing insights into the system’s behaviour, interactions amongst the systems’ variables, and governing principles are more paramount than just predictive outcomes. By revealing these dynamics, we can gain a deeper understanding of the system, which is pivotal for optimisation and scenario analysis.

In scenarios where the system’s dynamics can be adequately described using linear functions, the NARMAX model reduces to a linear model, the Autoregressive Moving Average with Exogenous Input (ARMAX). This adaptability ensures that NARMAX can describe a wide range of systems, from purely linear to highly nonlinear. The system’s description accuracy as well as the prediction accuracy of a NARMAX model, is dependent on the quality and representativeness of the data used during training. Nonetheless, unlike typical black-box approaches, NARMAX builds on a transparent structure based on differential or difference equations. This allows for a comprehensible interpretation of the model, enabling us to figure out key system characteristics and uncover dynamics that are generally hindered in deep learning models like LSTM.

In this study, we aim to investigate the connectedness between crypto assets and CO_2_ emissions. Additionally, we seek to compare the impact of both sustainable and conventional crypto assets on CO_2_ emissions. Since this is not a forecasting problem, deep learning models are not the preferred option. Although NARMAX is a data-driven modelling approach, it is considered a grey-box model because its terms explicitly reveal the dynamic elements within the system and their interrelationships, which drive variations in the output of interest. For this reason, NARMAX was chosen as the base model class for analysing both sustainable and conventional crypto assets. While forecasting CO_2_ emissions is not the primary focus of this study, we implemented an LSTM model on datasets for sustainable and conventional cryptocurrencies as a contemporary modelling paradigm for comparison.

#### NARMAX mathematical representation

A special class of NARMAX models, known as NARX, is often preferred in data modelling because estimating the Moving Average (MA) terms is not always necessary. The estimation of both the structure and parameters of a NARX model is significantly simpler compared to a NARMAX model. Typically, the structure of a NARX model is transparent, comprising differential and difference equations. A differential equation structure is preferred for continuous-time data, whereas for discrete signals, a difference equation representation is generally more appropriate within a typical NARX framework. NARX models can represent three primary classes of systems: Single Input Single Output (SISO), Multiple Inputs Single Output (MISO), and Multiple Inputs Multiple Outputs (MIMO).

A general representation of NARX is,

y(t)=F[y(t−1),y(t−2),…,y(t−k),x(t−d),x(t−d−1),…,x(t−d−l)]+e(t) (1)

where *y* (*t*) represents the output, *x* (*t*) stands for input, *e* (*t*) represents noise sequences, *k* is the maximum lag allowed for the system’s output, *l* is the maximum lag allowed for the system’s input, *F* is a function and *d* signifies the time delay.

The NARX model presented in Eq 1 can be simplified using the following linear-in-the-parameters version,

$$y(t)=\sum_{i=1}^{M}\theta_{i}\emptyset_{i}(t)+e(t)$$ (2)

In the linear-in-the-parameters representation (Eq 2), *y*(*t*) represents the output of the underlying system, with *t* = 1, 2,…,*N*. ∅_*i*_ is used for representing regressor terms with *i* = 1, 2,…,*M*, which are functions of the input, output, and their time-lagged values. These regressors can include linear and nonlinear combinations of time-lagged input and output variables. *θ*_*i*_ is the parameters set associated with each regressor, and *e*(*t*) signifies noise sequence. Since the parameters appear linearly in the linear-in-the-parameter form, standard linear regression techniques can be applied for estimating *θ*_*i*_. Regressors ∅_*i*_ capture nonlinear dynamics of the given system which allows the model to handle nonlinear complex behaviour whilst retaining the computational simplicity of the model. The linear-in-the-parameter form also makes it easier to interpret the contribution of each term to the output of interest, facilitating system analysis and understanding. The above linear-in-the-parameters version (Eq 2) can be transformed into,

y=∅θ+e (3)

where, $y={\left[y(1),y(2),\ldots ,y(N)\right]}^{T}$, ${\theta =[\theta_{1},\theta_{2},\ldots ,\theta_{M}]}^{T},{e=e(1),e(2),\ldots ,e(N)]}^{T}$, $\left[{\emptyset =\emptyset }_{1},\emptyset_{2},\ldots ,\emptyset_{M}\right]$.

The next step is to establish a ranking mechanism for the terms within the given NARX structure. The Forward Regression Orthogonal Least Squares (FROLS) method is widely used for this purpose. It utilises the Error Reduction Ratio (ERR) criterion to rank model terms based on their contribution to explaining the variance in the system’s output. ERR refers to the proportion of the total output variance that is accounted for by including a specific regressor to the model. It enables the quantification of the contribution of each candidate term to augment the model fit. It is therefore used in ranking terms during the iterative model structure determination process. A term having a higher ERR is prioritised. Ideally, the sum of all ERRs in a NARX model equals 1. However, it is uncommon to include all candidate terms in the model structure. Incorporating every term would result in an excessively large NARX model, increasing the likelihood of data overfitting. Consequently, selecting the optimal number of terms often requires domain-specific knowledge. In this study, FROLS was implemented using SysIdentPy [53].

#### Steps within NARX model identification

A typical Nonlinear System Identification consists of two major steps–model structure selection and parameter estimation. The model structure selection refers to the process of selecting only the most contributing terms in a model. In theory, all the possible terms depending on the maximum lag values of inputs, outputs, and degree of polynomial can be considered but that will not be considered an optimal model. In machine learning terminologies, we often call this scenario to be a type of overfitting. This means, that this kind of large model can show good accuracy over the training data but will most likely fail to predict the outputs satisfactorily over the test data. Hence, selecting optimal NARX model terms is a crucial step in a Nonlinear System Identification process. The FROLS algorithm is often used to rank the NARX model terms in order of their contribution to explain the variance in the output of interest. The ERR corresponding to every potential term within a NARX model is calculated using the FROLS algorithm, which helps in selecting the optimal number of terms. The sum of all the ERRs within a full NARX model should be equal to 1. However, as we intend to select only a few terms to avoid overfitting, the sum of ERRs is close to 1 for a good-fitted model but never exactly equal to 1.

This study has adopted the FROLS algorithm for model selection and parameter estimation. To run the FROLS algorithm and select the optimal number of terms in this study, a Python library, SysIdentPy [53] was employed. SysIdentPy is a user-friendly open-source Python package to perform NARX modelling on a given dataset. The package is capable of handling SISO and MISO NARX model identification, which satisfies our requirements. In this study, we aim to determine the connectedness between CO_2_ emissions and cryptocurrency trading volumes by revealing the dynamic relations between these variables. In Nonlinear System Identification terminology, we considered both SISO and MISO model structures for revealing the relations between CO_2_ emissions and the volume of the various types of cryptocurrencies.

#### NARX models development

In this study, we considered CO_2_ emissions as the output of interest. The linkage between emissions and various cryptocurrencies was analysed sequentially for both sustainable and conventional cryptocurrencies. The entire experiment was divided into two categories–sustainable cryptocurrency and conventional cryptocurrency. For each category, the SISO model corresponding to each of the available input variables was obtained but the output (CO_2_ emissions) was the same for all the SISO models. The degree of NARX polynomial was chosen to be 3 which allowed to capture most of the nonlinearities present in the data. Altogether, 50 regressor terms were selected for each of the SISO models and their goodness of fit was evaluated using standard metrics such as Root Mean Square Error (RMSE) and Mean Average Error (MAE). Similarly, SISO models corresponding to the sustainable cryptocurrency were obtained on a sequential basis. Considering the potential role of multiple cryptocurrencies on carbon emissions, we extended our study to the MISO where for both the categories of cryptocurrency, we obtained MISO models combining all the available input variables.

## Empirical results

### Connectedness between conventional crypto-assets and CO_2_ emissions

The results of the MISO–NARX model for conventional crypto-assets are reported in Table 2 based on the following econometric specification:

0.518.y(k−7)+0.487.y(k−14),…,−3.9933.x1(k−10).y(k−14) (4)

**Table 2:** Results for MISO–NARX model: Conventional crypto-assets.

Sr. #	Regressors	Parameters	ERR	Sr. #	Regressors	Parameters	ERR
0	y(k-7)	5.18E-01	6.91E-01	25	x5(k-2). y(k-12)	-3.11E+01	9.88E-04
1	y(k-14)	4.87E-01	5.56E-02	26	y(k-6)	1.48E-01	1.74E-03
2	x3(k-3). y(k-10)	-8.65E+01	7.98E-03	27	x3(k-11). y(k-13)	5.55E+01	1.24E-03
3	x1(k-7). x1(k-3)	-5.10E+01	4.62E-03	28	x2(k-6). y(k-3)	1.15E+01	9.85E-04
4	x5(k-2). x1(k-5)	-3.15E+01	4.43E-03	29	x4(k-14). y(k-4)	7.51E-01	1.06E-03
5	x2(k-14). y(k-15)	-2.45E+01	5.06E-03	30	x5(k-9). x1(k-5)	-6.44E+01	1.05E-03
6	y(k-1)	1.12E-01	4.54E-03	31	x1(k-1). y(k-12)	4.74E+01	1.02E-03
7	y(k-12). y(k-1)	-1.55E+00	5.33E-03	32	x5(k-14). y(k-3)	7.17E+01	8.41E-04
8	x3(k-1)	-4.43E+00	3.94E-03	33	x3(k-6). x1(k-2)	1.04E+01	7.64E-04
9	x4(k-11). y(k-5)	-3.90E+01	2.83E-03	34	x5(k-7). y(k-14)	5.81E+01	7.45E-04
10	x1(k-12)	-2.94E+00	3.71E-03	35	x2(k-14). y(k-1)	-2.88E+00	7.38E-04
11	x1(k-8). y(k-7)	-2.17E+01	2.78E-03	36	x5(k-14). y(k-8)	-2.39E+01	9.00E-04
12	x2(k-12). y(k-14)	-4.28E+01	2.53E-03	37	x4(k-12). x1(k-1)	-2.86E-01	8.08E-04
13	x3(k-6). x1(k-3)	-7.80E+00	2.13E-03	38	x5(k-4). x1(k-5)	6.78E+00	7.31E-04
14	y(k-10). y(k-8)	5.06E+01	1.78E-03	39	x3(k-6). y(k-14)	-4.21E+00	1.07E-03
15	x2(k-15)	-3.28E+00	1.40E-03	40	x5(k-7). y(k-11)	5.35E+01	8.28E-04
16	x4(k-1). y(k-6)	-6.57E+01	1.77E-03	41	y(k-14). y(k-4)	8.48E+01	7.55E-04
17	x4(k-5). y(k-2)	-7.73E+01	1.41E-03	42	x3(k-9). y(k-6)	5.61E+01	8.05E-04
18	y(k-15). y(k-13)	1.24E+02	1.18E-03	43	x1(k-10). y(k-4)	-5.94E+01	8.07E-04
19	x2(k-5). y(k-13)	-6.50E+01	2.46E-03	44	x3(k-7). y(k-8)	3.73E+00	6.52E-04
20	x1(k-1). y(k-15)	-7.38E+01	1.52E-03	45	y(k-6). y(k-5)	-2.92E+01	5.60E-04
21	x1(k-13). y(k-15)	4.81E+01	1.04E-03	46	x1(k-9). y(k-5)	-4.92E+01	5.96E-04
22	x5(k-1). y(k-13)	-2.91E+01	1.32E-03	47	x5(k-12). x1(k-6)	-3.16E-02	5.68E-04
23	x3(k-7). y(k-9)	-3.54E+01	9.99E-04	48	x3(k-6). y(k-5)	-5.04E+00	7.32E-04
24	y(k-4). y(k-3)	4.71E+01	1.13E-03	49	x1(k-10). y(k-14)	-3.99E+00	6.37E-04

Since the FROLS was used for obtaining the model terms, they are ranked based on the ERR. The term or regressor appearing first in the polynomial form of NARX (Eq 4) is the most contributing term towards the variance of the output of interest, which is CO_2_ emissions. Clearly, the first term is the autoregressive term consisting of time-lagged CO_2_ emissions. It indicates that the CO_2_ emissions on a given date are mostly dependent on the emission which was recorded a week ago. Despite the first two terms being entirely autoregressive, out of 50 terms in the NARX model, most of the terms consist of exogenous inputs. x1 represents BNB, x2, represents BTC, x3 represents ETH, x4 represents USDT, and x5 represents XRP. The maximum time lag in the NARX model corresponding to conventional cryptocurrencies is 15 days (about two weeks). This suggests that the state of crypto variables and the CO_2_ emissions variable can impact the state of CO_2_ emissions for up to two weeks. This also indicates the cascading nature of these variables. Altogether, 50 terms were selected in the model, but looking at the ERR values corresponding to the terms it is very obvious that only the first few terms or regressors are significant. However, the rest of the terms are still included in the model for better accuracy over the testing data.

The simulation of the NARX model corresponding to the conventional cryptocurrency is shown in S4 Fig. The simulation did not include training data, and the model was tested through fresh testing data.

To assess the sensitivity of each crypto variable towards the CO_2_ emissions, initially, the SISO NARX model corresponding to each crypto variable and CO_2_ emissions was developed. This allowed us to measure the degree of connectedness between a crypto variable and CO_2_ emissions by assuming that other crypto variables have no impact on CO_2_ emissions. Nonetheless, in the real world, all the cryptocurrencies are being mined simultaneously, and hence, the result of the MISO model should be deemed as a more realistic picture of the scenario.

In this study, Root Mean Square Error (RMSE) and MAE (Mean Average Error) are the chosen metrics to represent the goodness of fit of the model. Both RMSE and MAE are considered suitable options for measuring the goodness of fit in scenarios where the data is highly nonlinear and thus become the logical choice. The RMSE and MAE measures (Table 3) indicate that as the exogenous input variables like BNB, BTC, ETH, USDT, and XRP are added, the goodness of fit improves. In other words, the variance in CO_2_ emissions is greatly explained whilst all the candidate input variables are considered. The model structure (Table 2) and the associated Goodness of Fit measure (Table 3) demonstrate that there is a connectedness between crypto-assets and CO_2_ emissions. Hence, our first hypothesis is confirmed.

**Table 3:** Measures of forecast accuracy and goodness of fit for the proposed model, MISO–NARX: Conventional crypto-assets.

Input	RMSE	MAE
BNB	0.01952	0.01353
BTC	0.02136	0.01455
ETH	0.02750	0.01850
USDT	0.02737	0.01886
XRP	0.02196	0.01556
BNB, BTC	0.01874	0.01248
BNB, BTC, ETH	0.01888	0.01270
BNB, BTC, ETH, USDT	0.01962	0.01286
BNB, BTC, ETH, USDT, XRP	0.01962	0.01286

### Connectedness between sustainable crypto-assets and CO_2_ emissions

The results of the MISO–NARX model for sustainable crypto-assets are presented in Table 4 based on the following econometric specification:

0.548.y(k−7)+0.273.y(k−14),…,+36.2.x1(k−6).y(k−13) (5)

**Table 4:** Results for MISO–NARX model: Sustainable crypto-assets.

Sr. #	Regressors	Parameters	ERR	Sr. #	Regressors	Parameters	ERR
0	y(k-7)	5.48E-01	6.54E-01	25	x5(k-9). y(k-9)	8.20E+01	1.31E-03
1	y(k-14)	2.73E-01	4.35E-02	26	x3(k-11). y(k-10)	3.70E+01	1.07E-03
2	x5(k-5). y(k-9)	-1.46E+02	9.81E-03	27	x3(k-2). y(k-9)	-1.06E+02	1.20E-03
3	x5(k-6). y(k-14)	-4.51E+01	6.68E-03	28	x3(k-9). y(k-9)	1.06E+02	1.57E-03
4	x1(k-13). y(k-3)	6.23E+01	5.45E-03	29	x5(k-1). y(k-11)	-4.83E+00	1.31E-03
5	y(k-2)	-3.35E-01	9.03E-03	30	x4(k-3)	-2.66E+00	1.20E-03
6	y(k-4)	-2.04E-01	7.38E-03	31	x4(k-10). y(k-14)	-5.84E+01	1.03E-03
7	x2(k-6). y(k-11)	-2.57E+01	5.17E-03	32	x2(k-15). y(k-1)	1.24E-01	1.06E-03
8	x3(k-3)	4.29E+00	6.55E-03	33	y(k-3)^2	4.12E+01	1.00E-03
9	y(k-7). y(k-2)	-1.29E+00	6.64E-03	34	x3(k-8). y(k-2)	5.18E+01	2.18E-03
10	x1(k-12)	-3.48E+00	5.07E-03	35	x2(k-4). y(k-6)	-4.15E+01	1.02E-03
11	x3(k-4). y(k-14)	-5.71E+01	5.69E-03	36	x2(k-11).x1(k-2)	8.66E+00	1.03E-03
12	x4(k-7). x1(k-6)	1.63E-02	2.92E-03	37	x1(k-14). y(k-10)	-6.83E+00	1.22E-03
13	x2(k-3). y(k-7)	-4.43E+01	2.53E-03	38	x1(k-6). y(k-2)	-1.57E-01	1.02E-03
14	x5(k-12). x1(k-3)	-2.80E+01	2.39E-03	39	x1(k-9). y(k-14)	-3.50E+00	1.18E-03
15	x4(k-13). x1(k-5)	7.31E-02	2.16E-03	40	x1(k-15)	-1.35E+00	1.08E-03
16	x3(k-11). y(k-13)	6.13E+01	2.19E-03	41	x2(k-7). y(k-15)	5.03E+01	9.10E-04
17	y(k-9). y(k-7)	3.11E-01	1.93E-03	42	x5(k-11). y(k-12)	3.43E+01	1.43E-03
18	x1(k-4). y(k-10)	7.36E+00	2.23E-03	43	x4(k-4). y(k-15)	4.11E-02	9.80E-04
19	x1(k-3). y(k-13)	-1.95E+01	1.58E-03	44	x5(k-15). x1(k-4)	1.13E+00	1.25E-03
20	x3(k-5). y(k-13)	-5.84E+00	1.65E-03	45	x3(k-10). y(k-13)	-6.56E+01	1.06E-03
21	x1(k-7). y(k-10)	5.69E+01	1.32E-03	46	x2(k-9). y(k-14)	-7.35E-02	9.29E-04
22	x2(k-1). y(k-5)	-9.77E+01	1.44E-03	47	x4(k-8). x1(k-5)	1.02E-01	1.08E-03
23	x3(k-9). y(k-6)	9.14E+01	1.33E-03	48	x3(k-10). y(k-5)	3.40E+01	9.68E-04
24	x2(k-4). y(k-8)	6.06E+01	1.28E-03	49	x1(k-6). y(k-13)	3.62E+01	9.65E-04

Like conventional crypto, NARX models (Eq 5) for sustainable cryptocurrencies were developed in this study to perform a comparison of the connectedness with CO_2_ emissions for both types of cryptocurrencies. To perform a fair comparison 50 terms or regressors were considered like the conventional cryptocurrency category. Showing similarity to the conventional cryptocurrency, the first and the most contributing term is an auto-regressive one, y(k-7). This suggests that CO_2_ emissions on a certain day are best explained by the CO_2_ emissions recorded seven days before. The second term in the model is also an auto-regressive one suggesting that most of the dynamical variation in the CO_2_ emissions can be explained using auto-regressive terms.

Nonetheless, like the conventional crypto NARX model, the remaining terms do contain a substantial combination of other exogenous variables. The maximum time lag noticed in a few model terms is 15, which indicates that variables are contributing towards CO_2_ emissions for at least up to two weeks. The sum of ERR is 0.83373 when all the exogenous inputs are considered under conventional cryptocurrency NARX modelling. Whereas the sum of ERR is 0.81871 when all the exogenous inputs are considered under sustainable cryptocurrency NARX modelling. This indicates that input variables corresponding to the conventional cryptocurrency explain the variance in carbon emission slightly better as compared to the input variables corresponding to sustainable cryptocurrency. Hence, results merely support our hypothesis 2 that the effect of sustainable crypto-assets on CO_2_ emissions is different compared to conventional crypto-assets. The simulation of the NARX model corresponding to the sustainable cryptocurrency is shown in S5 Fig. The simulation is based on testing data.

In Table 5, Root Mean Square Error (RMSE), and Mean Absolute Error (MAE) corresponding to each input variable and a combination of those variables are presented.

**Table 5:** Measures of forecast accuracy and goodness of fit for the proposed model, MISO–NARX: Sustainable crypto-assets.

Input	RMSE	MAE
ADA	0.02286	0.01506
BITG	0.01951	0.01268
MIOTA	0.02035	0.01357
XNO	0.02439	0.01569
POWR	0.02220	0.01350
ADA, BITG	0.02130	0.01358
ADA, BITG, MIOTA	0.01967	0.01326
ADA, BITG, MIOTA, XNO	0.01946	0.01308
ADA, BITG, MIOTA, XNO, POWR	0.02046	0.01389

Again, the results based on different forecast accuracy measures show improvement in the goodness of fit of our proposed model. There are a few exceptions, which may be due to the random behaviour of the data. Finally, for brevity, the detailed results for the SISO–NARX model for individual conventional and sustainable crypto-assets are presented in Tables A1 to A10 in S1 Appendix.

### Comparison of NARX results with the Long Short-Term Memory (LSTM) model

The LSTM model has been extensively used for forecasting time series data [54–58]. An LSTM model setup is often employed for capturing underlying complex temporal relationships in time series data. This feature makes it appropriate for use cases requiring the prediction of output variables based on observed output variables and exogenous input variables. To generalise the model, regularisation through dropout is embedded in a typical LSTM fitting, which mitigates the risk of model overfitting, particularly for a deep network having multiple layers. In this study, LSTM was employed for both conventional and sustainable cryptocurrency data to compare the performance of the NARX with a contemporary machine-learning approach. Nonetheless, the aim of this research is mainly to find the connectedness between crypto trading and power consumption hence a transparent model structure such as NARX is more suitable than a black-box approach like LSTM. A typical deep learning approach like LSTM could be a better alternative in scenarios where forecasting the output of interest takes precedence over optimising the exogenous variables.

LSTM was first fitted for conventional cryptocurrency data. The input feature consisted of BNB-USD, BTC-USD, ETH-USD, USDT-USD, XRP-USD, 1^st^ lag value of Power, 2^nd^ lag value of Power, whereas the output variable was Power. Min-Max scaler was used for scaling both the input variable set and the output variable. The data preparation for the LSTM model took place using a sliding window technique having a time step equal to 7. The data was split into training and testing, like the NARX modelling, where 85% of the data points were used for training, and the remaining 15% of the data were kept aside for model validation. TensorFlow Keras Sequential API was used for constructing a multi-layer LSTM model. The LSTM architecture consisted of five layers, each of the layers was followed by the dropout layers to minimise overfitting. The final layer was a fully connected layer having a single unit which generates the prediction of the output variable at a given time step. The LSTM layers in the model use the default internal activation function, tanh, and the default gate activation function, sigmoid. The architecture comprises five LSTM layers with 350, 300, 250, 200, and 150 nodes, respectively, in the first to fifth layers. A dropout rate of 0.3 is applied after each LSTM layer to mitigate overfitting. The model is optimised using the Adam optimiser, chosen for its adaptive learning rate capabilities. Adam is considered appropriate for such types of continuous variable data. The mean squared error is used as the loss function to minimise prediction errors. The model is trained over 40 epochs with a batch size of 32.

The model simulation results based on the testing data are shown in S6 Fig, exhibiting a similar level of accuracy for both the LSTM and NARX models. However, the error metrics reported in Table 6 indicate that the NARX model performed slightly better than the LSTM model. Although this performance difference is small, it could be due to several underlying factors. Firstly, the NARX model structure explicitly included lagged input data while constituting regressor terms, which improves its ability to capture time dependencies in the data. Secondly, NARX models are simpler and generally have fewer trainable parameters compared to LSTM models, which mitigates the risk of overfitting, especially in this case when the dataset size is limited. This feature allows NARX models to generalise better to unseen data. Furthermore, LSTM model architectures that rely on memory cells and gates to capture long-term dependencies may not offer significant advantages in this context, where short-term relationships amongst the variables appear to be more influential. While the performance differences are marginal between NARX and LSTM, the NARX model’s interpretability and its ability to exhibit the level of connectedness among crypto trading values and carbon emissions further suggest that NARX is a more suitable model class in this study.

**Table 6:** Measures of forecast accuracy and goodness of fit for the proposed model, LSTM: Conventional crypto-assets.

Mean Absolute Error (MAE)	0.01527
Root Mean Squared Error (RMSE)	0.02284

Similarly, LSTM was fitted for sustainable cryptocurrency data. The input feature consisted of ADA-USD, BITG-USD, MIOTA-USD, XNO-USD, POWR-USD, 1^st^ lag value of Power, 2^nd^ lag value of Power, whereas the output variable was Power. The LSTM simulation results based on the testing data kept aside for validation are shown in S7 Fig. The plot shows a reasonable level of accuracy and is very much similar to the NARX model. Nevertheless, the error metrics reported in Table 7 suggest that the NARX model performed slightly better than LSTM. As explained above, irrespective of nearly similar error metrics and simulation results, the NARX is a more suitable option to understand the connectedness among the crypto trading values and the associated carbon emission.

**Table 7:** Measures of forecast accuracy and goodness of fit for the proposed model, LSTM: Sustainable crypto-assets.

Mean Absolute Error (MAE)	0.01468
Root Mean Squared Error (RMSE)	0.02063

## Conclusion

The objective of this research was to propose a Nonlinear System Identification data-driven technique for the crypto market and CO_2_ emissions nexus, utilising machine learning algorithms, specifically the Multiple Inputs Single Output (MISO) Nonlinear Autoregressive Network with Exogenous Inputs (NARX). For the application of the Nonlinear System Identification model, MISO–NARX, we considered the daily data of conventional crypto-assets, sustainable crypto-assets, and CO_2_ emissions from January 2, 2019, to March 31, 2023. In this study, a parsimonious polynomial NARX model was developed to predict the dynamic responses of CO_2_ emissions (5 inputs and 1 output) using the FROLS algorithm. The FROLS algorithm proved to be a powerful tool in selecting the most significant model terms for representing the dynamical connectedness of the response variables (CO_2_ emissions) and the exogenous variables corresponding to both conventional and sustainable crypto-assets.

In a nutshell, our findings indicate that in the case of the connectedness of the crypto market and CO_2_ emissions, conventional crypto-assets show slightly stronger connections in comparison to sustainable crypto-assets. Conventional crypto-assets have a stronger environmental footprint in terms of CO_2_ emissions due to the energy consumption of PoW-based mining, compared to more sustainable crypto-assets that rely on less energy-intensive methods. Specifically, conventional crypto-assets use PoW as their consensus mechanism. In PoW, miners compete to solve complex cryptographic puzzles using vast amounts of computational power. This process requires significant energy, much of which is still derived from fossil fuels, especially in regions where electricity is cheap but carbon-intensive, such as coal-powered grids. As a result, mining activities for these cryptocurrencies lead to higher CO_2_ emissions. In contrast, sustainable crypto-assets typically use PoS or other environmentally friendly consensus mechanisms, like proof of authority (PoA) or delegated proof of stake (DPoS). PoS, for example, does not require the same computational work as PoW and relies on participants who hold and lock up cryptocurrency to validate transactions, making it much less energy-intensive. Additionally, some sustainable crypto projects are designed to offset their carbon footprint through green initiatives or by using renewable energy sources for mining or transaction processing. The reduced energy consumption directly correlates with lower CO_2_ emissions.

Our results imply that, to a certain extent, sustainable crypto-assets offer a potential solution to environmental challenges, and our findings align with global sustainability goals such as the Paris Agreement in the following ways. The Paris Agreement aims to limit global warming to below 2°C, preferably to 1.5°C, by reducing greenhouse gas emissions. As cryptocurrencies transition toward sustainable crypto-assets using more energy-efficient technologies, they can contribute to these goals by lowering their carbon footprint. For example, networks that shift to PoS or use renewable energy can significantly reduce their overall environmental impact. Additionally, the decentralised finance (DeFi) sector of crypto-assets provides financial services without the need for traditional banking infrastructure, thus reducing the energy and resource demands of the conventional financial system. By operating on more energy-efficient networks, sustainable DeFi tokens can align with sustainable finance initiatives that promote climate action and responsible consumption, both of which are key aspects of the United Nations Sustainable Development Goals. Nonetheless, there is a need for further improvements in sustainable crypto-assets through technological advancements to create more energy-efficient decentralised finance consensus algorithms to transform the cryptocurrency ecosystem into an environmentally sustainable market.

Our model shows good predictability power; however, we used the trading volumes of crypto-assets as a measure of crypto-trading and linked with CO_2_ emissions. In future studies, researchers can use our proposed model based on machine learning algorithms and test the connectedness of electricity consumption through the mining of specific crypto-assets and CO_2_ emissions. For example, researchers can use Cambridge University’s Bitcoin and Ethereum Electricity Consumption Index as a proxy for crypto-assets mining and explore its connectedness with CO_2_ emissions. This will further refine our findings as well as the findings of Zhang et al. [14]. Moreover, future research studies can also explore whether the connection between cryptocurrencies and CO_2_ emissions for countries having crypto-mining units differs from those having no crypto-mining units. We leave these issues for future research.

## Supporting information

S1 Fig Log transformed CO_2_ from January 2, 2019, to March 31, 2023. Source: Authors’ calculations and graphical adjustments using Carbon Monitor online data.
(TIF)

S2 Fig Log transformed daily trading volumes of conventional crypto-assets from January 2, 2019, to March 31, 2023. Source: Authors’ calculations and graphical adjustments using Coinmarket cap.com and Yahoo Finance online data.
(TIF)

S3 Fig Log transformed daily trading volumes of sustainable crypto-assets from January 2, 2019, to March 31, 2023. Source: Authors’ calculations and graphical adjustments using Coinmarket cap.com and Yahoo Finance online data.
(TIF)

S4 Fig The simulation of the NARX model corresponding to the conventional crypto-assets. Source: Authors’ calculations and graphical adjustments using Carbon Monitor, Coinmarket cap.com, and Yahoo Finance online data.
(TIF)

S5 Fig The simulation of the NARX model corresponding to the sustainable crypto-assets. Source: Authors’ calculations and graphical adjustments using Carbon Monitor, Coinmarket cap.com, and Yahoo Finance online data.
(TIF)

S6 Fig The simulation of the LSTM corresponding to the conventional crypto-assets. Source: Authors’ calculations and graphical adjustments using Carbon Monitor, Coinmarket cap.com, and Yahoo Finance online data.
(TIF)

S7 Fig The simulation of the LSTM corresponding to the sustainable crypto-assets. Source: Authors’ calculations and graphical adjustments using Carbon Monitor, Coinmarket cap.com, and Yahoo Finance online data.
(TIF)

S1 Appendix APPENDIX A: Results of SISO–NARX model for individual conventional and sustainable crypto-assets. (DOCX)

S1 Data (XLSX)
