# Supplementary figures and images for: Nonlinear connectedness of conventional crypto-assets and sustainable crypto-assets with climate change: A complex systems modelling approach
Source: PLoS One. 2025 Feb 7;20(2):e0318647. doi: 10.1371/journal.pone.0318647 (PMC11805393; doi:10.1371/journal.pone.0318647)

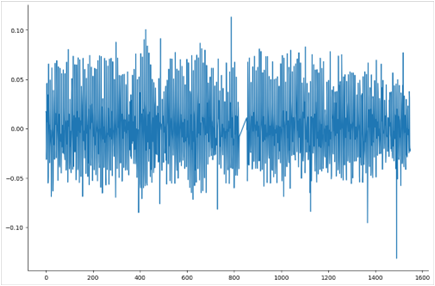

Supplement: S1 Fig — Source: Authors’ calculations and graphical adjustments using Carbon Monitor online data. (TIF) [file pone.0318647.s001.tif]

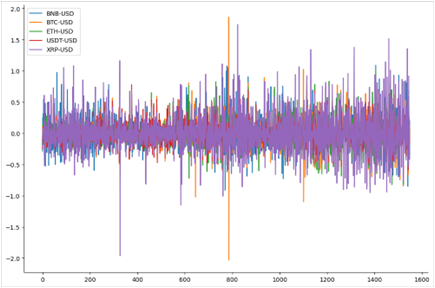

Supplement: S2 Fig — Source: Authors’ calculations and graphical adjustments using Coinmarket cap.com and Yahoo Finance online data. (TIF) [file pone.0318647.s002.tif]

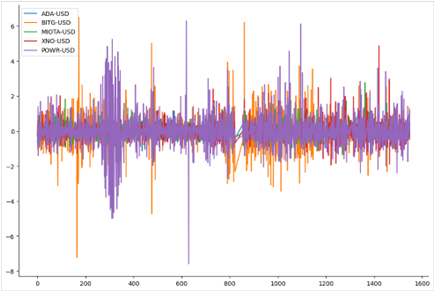

Supplement: S3 Fig — Source: Authors’ calculations and graphical adjustments using Coinmarket cap.com and Yahoo Finance online data. (TIF) [file pone.0318647.s003.tif]

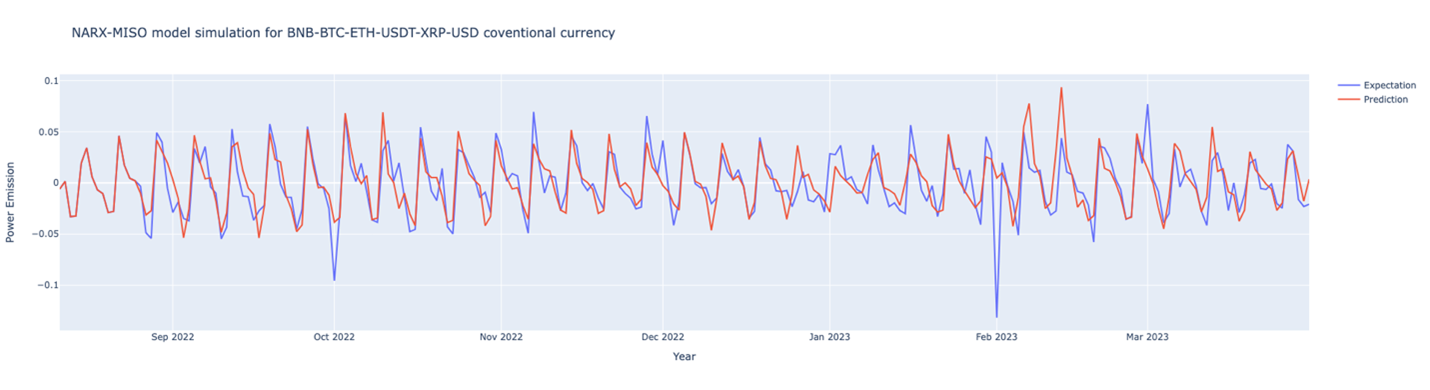

Supplement: S4 Fig — Source: Authors’ calculations and graphical adjustments using Carbon Monitor, Coinmarket cap.com, and Yahoo Finance online data. (TIF) [file pone.0318647.s004.tif]

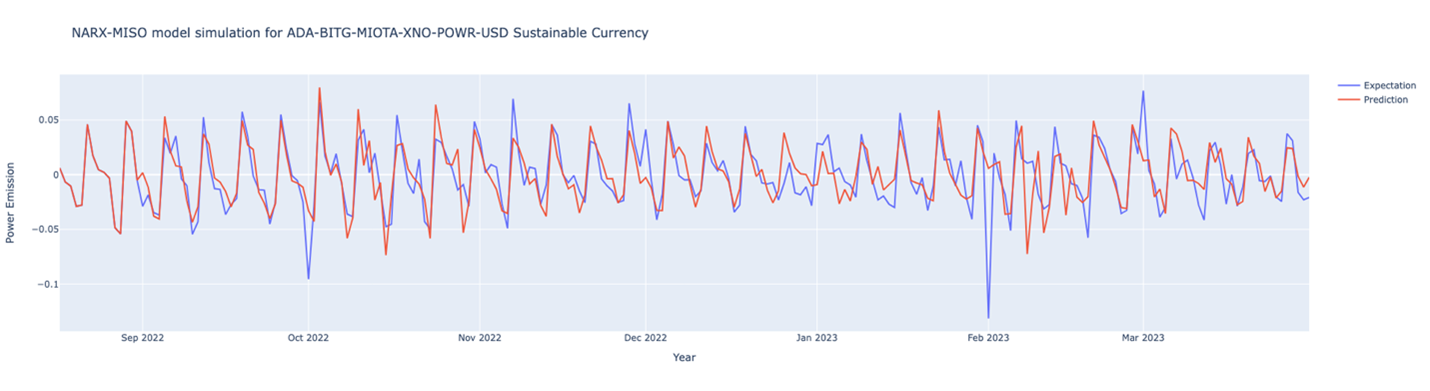

Supplement: S5 Fig — Source: Authors’ calculations and graphical adjustments using Carbon Monitor, Coinmarket cap.com, and Yahoo Finance online data. (TIF) [file pone.0318647.s005.tif]

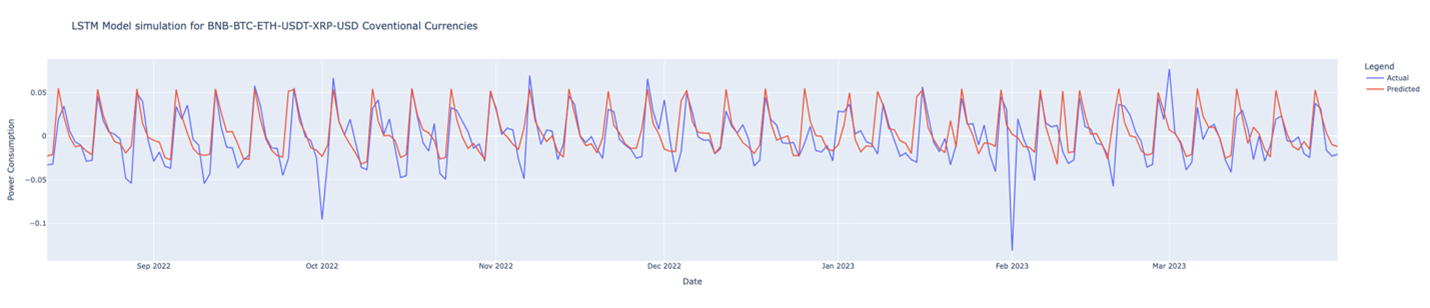

Supplement: S6 Fig — Source: Authors’ calculations and graphical adjustments using Carbon Monitor, Coinmarket cap.com, and Yahoo Finance online data. (TIF) [file pone.0318647.s006.tif]

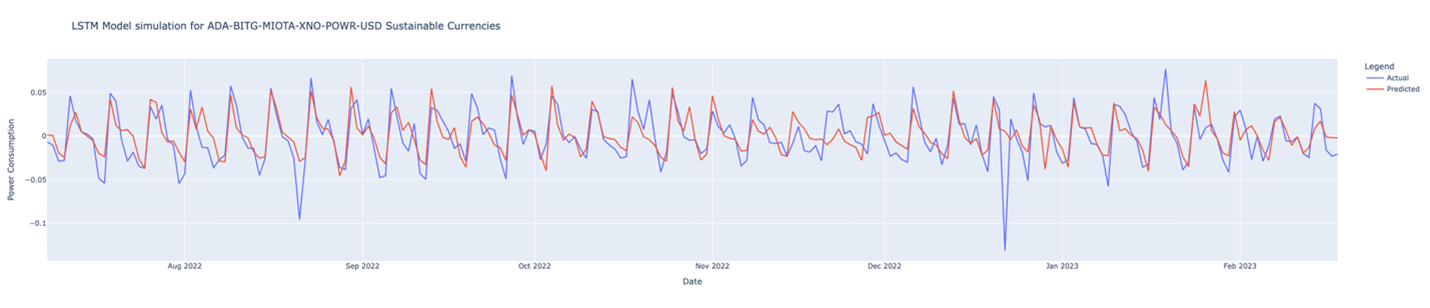

Supplement: S7 Fig — Source: Authors’ calculations and graphical adjustments using Carbon Monitor, Coinmarket cap.com, and Yahoo Finance online data. (TIF) [file pone.0318647.s007.tif]
