# Supplementary material for: Nonlinear connectedness of conventional crypto-assets and sustainable crypto-assets with climate change: A complex systems modelling approach
Source: PLoS One. 2025 Feb 7;20(2):e0318647. doi: 10.1371/journal.pone.0318647 (PMC11805393; doi:10.1371/journal.pone.0318647)
Supplement: S1 Appendix — (DOCX) [file pone.0318647.s008.docx]

**APPENDIX A**

**Results of SISO–NARX model for individual conventional and sustainable crypto-assets.**

| **Table A1: BNB-USD Conventional** | | | | | | | |
| --- | --- | --- | --- | --- | --- | --- | --- |
| **Sr. #** | **Regressors** | **Parameters** | **ERR** | **Sr. #** | **Regressors** | **Parameters** | **ERR** |
| 0 | y(k-7) | 0.463 | 0.691 | 25 | x1(k-13)^2y(k-2) | -0.354 | 0.00142 |
| 1 | y(k-14) | 0.456 | 0.0556 | 26 | x1(k-11)x1(k-3)y(k-3) | -0.719 | 0.0014 |
| 2 | y(k-14)y(k-11)y(k-4) | -107 | 0.00798 | 27 | x1(k-7)^3 | 0.0142 | 0.00143 |
| 3 | y(k-14)y(k-10)^2 | -8.43 | 0.00462 | 28 | x1(k-4)x1(k-2) | -0.0206 | 0.00123 |
| 4 | y(k-14)^3 | -35.7 | 0.00443 | 29 | y(k-11)^2y(k-7) | -61.8 | 0.0012 |
| 5 | y(k-15)y(k-14)y(k-7) | -61.4 | 0.00506 | 30 | x1(k-14)y(k-4) | 0.132 | 0.00116 |
| 6 | y(k-1) | 0.115 | 0.00454 | 31 | y(k-4) | -0.0821 | 0.00111 |
| 7 | y(k-12)y(k-5) | -1.97 | 0.00533 | 32 | x1(k-11)y(k-6)y(k-2) | -7.53 | 0.0011 |
| 8 | y(k-7)y(k-2) | -3.64 | 0.00394 | 33 | y(k-7)y(k-3)y(k-2) | -75.9 | 0.00107 |
| 9 | y(k-6)^2y(k-2) | -35.6 | 0.00283 | 34 | x1(k-13)x1(k-3)y(k-5) | 0.666 | 0.00102 |
| 10 | y(k-7)y(k-1) | -2.93 | 0.00371 | 35 | y(k-12)y(k-6) | 1.23 | 0.000956 |
| 11 | y(k-4)y(k-3)^2 | -15.2 | 0.00278 | 36 | y(k-2) | -0.0804 | 0.00107 |
| 12 | y(k-7)^3 | -30.3 | 0.00253 | 37 | y(k-11)y(k-9)y(k-1) | -55.2 | 0.00125 |
| 13 | x1(k-15)y(k-13)y(k-10) | -9.79 | 0.00213 | 38 | x1(k-10)x1(k-9)y(k-15) | 0.627 | 0.00101 |
| 14 | x1(k-12)x1(k-10)^2 | 0.0329 | 0.00195 | 39 | x1(k-9)y(k-7)y(k-2) | -4.52 | 0.00102 |
| 15 | y(k-12)y(k-7)y(k-3) | 33 | 0.00177 | 40 | x1(k-8)y(k-14) | 0.146 | 0.000925 |
| 16 | y(k-6)y(k-2) | -2.74 | 0.00157 | 41 | x1(k-3)y(k-14)y(k-10) | -10.3 | 0.00104 |
| 17 | y(k-12)^2y(k-2) | -39.2 | 0.00163 | 42 | x1(k-15)x1(k-4)y(k-3) | -0.516 | 0.000936 |
| 18 | x1(k-14)x1(k-7)y(k-10) | -0.65 | 0.00152 | 43 | y(k-10)y(k-7)^2 | 44.2 | 0.000925 |
| 19 | x1(k-14)x1(k-1)y(k-2) | 0.513 | 0.00148 | 44 | y(k-14)y(k-9) | -1.87 | 0.000893 |
| 20 | x1(k-13)y(k-12) | -0.235 | 0.00149 | 45 | x1(k-13)x1(k-12)x1(k-3) | -0.0627 | 0.000878 |
| 21 | y(k-15)^2y(k-14) | -56.8 | 0.00152 | 46 | x1(k-12)x1(k-6)y(k-6) | -0.518 | 0.000929 |
| 22 | y(k-15)y(k-6)^2 | 53.5 | 0.0015 | 47 | y(k-14)y(k-11)y(k-3) | 63.8 | 0.000794 |
| 23 | y(k-8)y(k-7)y(k-6) | 62.7 | 0.00225 | 48 | x1(k-10)x1(k-3)y(k-14) | 0.542 | 0.000804 |
| 24 | x1(k-11)y(k-10)y(k-6) | -7.66 | 0.00147 | 49 | x1(k-4)y(k-14)y(k-10) | -6.73 | 0.000857 |

| **Table A2: BTC-USD Conventional** | | | | | | | |
| --- | --- | --- | --- | --- | --- | --- | --- |
| **Sr. #** | **Regressors** | **Parameters** | **ERR** | **Sr. #** | **Regressors** | **Parameters** | **ERR** |
| 0 | y(k-7) | 0.392 | 0.691 | 25 | y(k-7)y(k-3)y(k-2) | -57.2 | 0.0014 |
| 1 | y(k-14) | 0.488 | 0.0556 | 26 | y(k-11)^2y(k-7) | -34.6 | 0.00142 |
| 2 | y(k-14)y(k-11)y(k-4) | -92.6 | 0.00798 | 27 | x1(k-8)x1(k-3)y(k-13) | 0.631 | 0.00124 |
| 3 | y(k-14)y(k-10)^2 | -54.2 | 0.00462 | 28 | x1(k-13)x1(k-1)y(k-7) | 0.621 | 0.00123 |
| 4 | y(k-14)^3 | -33.4 | 0.00443 | 29 | x1(k-13)y(k-15) | 0.231 | 0.0014 |
| 5 | y(k-15)y(k-14)y(k-7) | -42.3 | 0.00506 | 30 | x1(k-12)x1(k-5)y(k-8) | 0.777 | 0.00143 |
| 6 | y(k-1) | 0.14 | 0.00454 | 31 | x1(k-5)y(k-7)y(k-4) | -9.62 | 0.00108 |
| 7 | y(k-12)y(k-5) | -2.2 | 0.00533 | 32 | y(k-6)y(k-2) | -1.97 | 0.00104 |
| 8 | y(k-7)y(k-2) | -3.5 | 0.00394 | 33 | x1(k-7)x1(k-5)y(k-11) | 0.583 | 0.000987 |
| 9 | x1(k-11)x1(k-2)^2 | -0.00609 | 0.00371 | 34 | x1(k-12)y(k-13)y(k-5) | -7.02 | 0.000981 |
| 10 | y(k-6)^2y(k-2) | -75.5 | 0.00278 | 35 | y(k-14)y(k-13)y(k-5) | -59.1 | 0.00109 |
| 11 | y(k-7)y(k-1) | -3.1 | 0.00352 | 36 | x1(k-12)x1(k-7)x1(k-1) | -0.105 | 0.000981 |
| 12 | y(k-4)y(k-3)^2 | -27.8 | 0.0026 | 37 | x1(k-5)y(k-15)y(k-4) | 9.61 | 0.000914 |
| 13 | y(k-7)^3 | -25.5 | 0.00244 | 38 | x1(k-11)x1(k-9)x1(k-5) | -0.101 | 0.00115 |
| 14 | x1(k-7)x1(k-2)y(k-12) | -1.05 | 0.00169 | 39 | x1(k-12)y(k-15)y(k-4) | -8.73 | 0.00098 |
| 15 | x1(k-6)x1(k-2)y(k-10) | 0.689 | 0.00192 | 40 | x1(k-12)y(k-10)y(k-7) | 6.6 | 0.00113 |
| 16 | x1(k-14)y(k-1) | -0.187 | 0.00172 | 41 | x1(k-12)x1(k-2)y(k-2) | -0.596 | 0.00112 |
| 17 | y(k-6)^2y(k-5) | -48.8 | 0.00173 | 42 | y(k-14)y(k-7) | 1.87 | 0.00108 |
| 18 | x1(k-12)x1(k-9)x1(k-8) | -0.0749 | 0.00169 | 43 | y(k-13)y(k-9)y(k-6) | 50.8 | 0.00102 |
| 19 | y(k-6)y(k-4) | 2.12 | 0.00168 | 44 | x1(k-8)y(k-13)y(k-3) | 8.71 | 0.00103 |
| 20 | y(k-15)^2y(k-14) | -43.2 | 0.0015 | 45 | x1(k-7)y(k-5)y(k-1) | -7.46 | 0.000983 |
| 21 | y(k-12)^2y(k-2) | -54.4 | 0.00158 | 46 | x1(k-14)y(k-14)y(k-4) | -7.86 | 0.000914 |
| 22 | x1(k-11)y(k-13)y(k-3) | -4.6 | 0.00144 | 47 | x1(k-15)y(k-13)y(k-10) | -5.51 | 0.00105 |
| 23 | x1(k-11)x1(k-8)y(k-3) | 1.29 | 0.00196 | 48 | x1(k-7)x1(k-3)y(k-7) | 0.534 | 0.0009 |
| 24 | x1(k-6)x1(k-2)y(k-2) | 0.81 | 0.00147 | 49 | x1(k-10)x1(k-8)y(k-9) | 0.606 | 0.000928 |

| **Table A3: ETH-USD Conventional** | | | | | | | |
| --- | --- | --- | --- | --- | --- | --- | --- |
| **Sr. #** | **Regressors** | **Parameters** | **ERR** | **Sr. #** | **Regressors** | **Parameters** | **ERR** |
| 0 | y(k-7) | 0.421 | 0.691 | 25 | x1(k-12)y(k-13)y(k-2) | 8.74 | 0.0015 |
| 1 | y(k-14) | 0.425 | 0.0556 | 26 | x1(k-11)x1(k-9)x1(k-5) | -0.0558 | 0.00137 |
| 2 | y(k-14)y(k-11)y(k-4) | -74.7 | 0.00798 | 27 | x1(k-5)x1(k-1)y(k-7) | -0.779 | 0.00133 |
| 3 | y(k-14)y(k-10)^2 | -39.1 | 0.00462 | 28 | x1(k-6)y(k-7)^2 | 3.78 | 0.00125 |
| 4 | y(k-14)^3 | -26 | 0.00443 | 29 | y(k-11)y(k-9)y(k-1) | -70 | 0.00119 |
| 5 | y(k-15)y(k-14)y(k-7) | -55.1 | 0.00506 | 30 | y(k-2) | -0.145 | 0.00216 |
| 6 | y(k-1) | 0.123 | 0.00454 | 31 | y(k-4) | -0.13 | 0.00128 |
| 7 | y(k-12)y(k-5) | -2.4 | 0.00533 | 32 | y(k-13)y(k-10)y(k-6) | 47.9 | 0.00164 |
| 8 | y(k-7)y(k-2) | -2.3 | 0.00394 | 33 | x1(k-13)x1(k-8)^2 | -0.0435 | 0.0014 |
| 9 | x1(k-11)x1(k-8)x1(k-7) | 0.165 | 0.00362 | 34 | x1(k-7) | 0.00859 | 0.00131 |
| 10 | y(k-6)^2y(k-2) | -21.4 | 0.00292 | 35 | x1(k-7)y(k-8)y(k-6) | 7.32 | 0.00112 |
| 11 | y(k-7)y(k-1) | -2.98 | 0.00354 | 36 | x1(k-12)x1(k-7)y(k-12) | -0.659 | 0.00131 |
| 12 | y(k-4)y(k-3)^2 | -10.2 | 0.00275 | 37 | y(k-6)y(k-2) | -2.61 | 0.00124 |
| 13 | x1(k-11)x1(k-9)x1(k-8) | 0.129 | 0.00247 | 38 | x1(k-11)x1(k-5)y(k-8) | -1.04 | 0.00113 |
| 14 | y(k-7)^3 | -22.9 | 0.00267 | 39 | y(k-9)y(k-6)y(k-4) | -59.2 | 0.00114 |
| 15 | y(k-11)^2y(k-7) | -58.1 | 0.00176 | 40 | x1(k-14)y(k-15) | -0.184 | 0.00113 |
| 16 | x1(k-8)x1(k-4)x1(k-2) | -0.0848 | 0.00167 | 41 | x1(k-6)x1(k-4)y(k-1) | 0.714 | 0.00115 |
| 17 | x1(k-12)x1(k-9)x1(k-6) | -0.11 | 0.00168 | 42 | x1(k-10)x1(k-4)y(k-13) | -0.93 | 0.00118 |
| 18 | y(k-7)y(k-5)y(k-3) | 55.6 | 0.00186 | 43 | x1(k-5)y(k-8)y(k-4) | 13.4 | 0.00115 |
| 19 | x1(k-9)y(k-15)y(k-14) | -4.79 | 0.00166 | 44 | y(k-15)y(k-14)y(k-8) | -49.1 | 0.00113 |
| 20 | x1(k-11)x1(k-6) | -0.0301 | 0.00154 | 45 | y(k-15)y(k-6)^2 | 28.7 | 0.00133 |
| 21 | x1(k-12)y(k-15)y(k-4) | -9.81 | 0.00142 | 46 | y(k-7)^2y(k-4) | 45.5 | 0.00105 |
| 22 | x1(k-8)x1(k-3)y(k-13) | 0.791 | 0.00149 | 47 | x1(k-5)y(k-4)y(k-1) | -8.6 | 0.0011 |
| 23 | x1(k-13)y(k-15) | 0.241 | 0.00147 | 48 | x1(k-2)y(k-14)y(k-9) | 3.99 | 0.000871 |
| 24 | x1(k-13)y(k-14)y(k-13) | -6.03 | 0.00161 | 49 | y(k-12)y(k-6) | 1.17 | 0.000858 |

| **Table A4: USDT-USD Conventional** | | | | | | | |
| --- | --- | --- | --- | --- | --- | --- | --- |
| **Sr. #** | **Regressors** | **Parameters** | **ERR** | **Sr. #** | **Regressors** | **Parameters** | **ERR** |
| 0 | y(k-7) | 0.455 | 0.691 | 25 | y(k-12)^2y(k-2) | -68.9 | 0.00132 |
| 1 | y(k-14) | 0.465 | 0.0556 | 26 | y(k-6)y(k-2) | -2.85 | 0.00163 |
| 2 | y(k-14)y(k-11)y(k-4) | -103 | 0.00798 | 27 | x1(k-3)y(k-9)^2 | 4.07 | 0.00151 |
| 3 | y(k-14)y(k-10)^2 | -44.6 | 0.00462 | 28 | y(k-12)y(k-7)y(k-6) | -55.1 | 0.00136 |
| 4 | y(k-14)^3 | -21.5 | 0.00443 | 29 | y(k-4) | -0.0983 | 0.00162 |
| 5 | y(k-15)y(k-14)y(k-7) | -39.1 | 0.00506 | 30 | y(k-15)y(k-8)y(k-6) | -71.1 | 0.00139 |
| 6 | y(k-1) | 0.118 | 0.00454 | 31 | y(k-8)y(k-7)y(k-6) | 76.6 | 0.00141 |
| 7 | y(k-12)y(k-5) | -2.05 | 0.00533 | 32 | y(k-7)y(k-4)^2 | -47.1 | 0.00154 |
| 8 | y(k-7)y(k-2) | -3.64 | 0.00394 | 33 | y(k-15)^2y(k-14) | -48.5 | 0.0014 |
| 9 | x1(k-11)x1(k-8)x1(k-7) | 0.191 | 0.00346 | 34 | y(k-14)y(k-12)y(k-7) | 60.8 | 0.00128 |
| 10 | y(k-6)^2y(k-2) | -28.8 | 0.00291 | 35 | x1(k-7)y(k-6)y(k-1) | 6.59 | 0.00127 |
| 11 | y(k-7)y(k-1) | -3.62 | 0.00365 | 36 | x1(k-13)x1(k-11)x1(k-2) | -0.133 | 0.00126 |
| 12 | y(k-4)y(k-3)^2 | -15.2 | 0.0029 | 37 | x1(k-2)y(k-6)y(k-2) | 8.02 | 0.0012 |
| 13 | y(k-7)^3 | -25.8 | 0.0024 | 38 | x1(k-12)x1(k-6)x1(k-4) | -0.148 | 0.00104 |
| 14 | y(k-7)y(k-5)y(k-3) | 40.5 | 0.00179 | 39 | x1(k-10)y(k-15) | -0.177 | 0.00106 |
| 15 | x1(k-12)x1(k-9)x1(k-5) | 0.179 | 0.00174 | 40 | x1(k-12)y(k-10)y(k-7) | 7.34 | 0.00101 |
| 16 | x1(k-12)x1(k-11)x1(k-8) | 0.199 | 0.00153 | 41 | x1(k-5)y(k-7)y(k-4) | -17.5 | 0.00112 |
| 17 | x1(k-14)x1(k-11)x1(k-8) | 0.202 | 0.00161 | 42 | x1(k-5)y(k-8)y(k-4) | 12.3 | 0.00108 |
| 18 | x1(k-5)^2y(k-9) | -0.729 | 0.00151 | 43 | x1(k-5)y(k-6)y(k-4) | 7.49 | 0.00129 |
| 19 | x1(k-15)x1(k-14)x1(k-5) | 0.155 | 0.00161 | 44 | x1(k-4)y(k-10)y(k-2) | -6.38 | 0.00127 |
| 20 | y(k-13)y(k-10)y(k-6) | 63.6 | 0.00167 | 45 | y(k-14)y(k-11)y(k-5) | 55.8 | 0.00105 |
| 21 | x1(k-15)^2x1(k-7) | 0.102 | 0.00171 | 46 | x1(k-5)y(k-13)y(k-7) | -5.88 | 0.00107 |
| 22 | x1(k-12)y(k-7)y(k-6) | 6.5 | 0.00151 | 47 | x1(k-8)x1(k-7)x1(k-3) | -0.13 | 0.000982 |
| 23 | x1(k-12)x1(k-9)x1(k-6) | -0.184 | 0.00171 | 48 | x1(k-6)y(k-15)y(k-2) | 5.09 | 0.000887 |
| 24 | x1(k-14)y(k-2)y(k-1) | 7.39 | 0.00149 | 49 | y(k-14)y(k-11)y(k-3) | 69.1 | 0.000925 |

| **Table A5: XRP-USD Conventional** | | | | | | | |
| --- | --- | --- | --- | --- | --- | --- | --- |
| **Sr. #** | **Regressors** | **Parameters** | **ERR** | **Sr. #** | **Regressors** | **Parameters** | **ERR** |
| 0 | y(k-7) | 0.366 | 0.691 | 25 | x1(k-11)x1(k-9)x1(k-6) | 0.0378 | 0.00136 |
| 1 | y(k-14) | 0.511 | 0.0556 | 26 | x1(k-7)x1(k-4)y(k-7) | -0.398 | 0.00125 |
| 2 | y(k-14)y(k-11)y(k-4) | -116 | 0.00798 | 27 | x1(k-12)x1(k-5)y(k-14) | -0.534 | 0.00137 |
| 3 | y(k-14)y(k-10)^2 | -48.9 | 0.00462 | 28 | y(k-12)y(k-8)y(k-2) | 63.5 | 0.00131 |
| 4 | y(k-14)^3 | -24 | 0.00443 | 29 | y(k-15)^2y(k-14) | -42.9 | 0.0015 |
| 5 | y(k-15)y(k-14)y(k-7) | -39.6 | 0.00506 | 30 | x1(k-2)y(k-14) | -0.104 | 0.00128 |
| 6 | y(k-1) | 0.121 | 0.00454 | 31 | x1(k-11)y(k-14)y(k-5) | 3.04 | 0.00125 |
| 7 | y(k-12)y(k-5) | -1.67 | 0.00533 | 32 | x1(k-11)x1(k-9)y(k-2) | -0.38 | 0.00112 |
| 8 | y(k-7)y(k-2) | -3.38 | 0.00394 | 33 | x1(k-10)x1(k-1)y(k-4) | 0.458 | 0.00114 |
| 9 | y(k-6)^2y(k-2) | -39 | 0.00283 | 34 | x1(k-6)x1(k-1)y(k-2) | 0.458 | 0.0011 |
| 10 | y(k-7)y(k-1) | -4.29 | 0.00371 | 35 | x1(k-10)x1(k-4)y(k-12) | 0.613 | 0.00115 |
| 11 | y(k-4)y(k-3)^2 | -24.9 | 0.00278 | 36 | x1(k-14)x1(k-10)y(k-5) | -0.446 | 0.00107 |
| 12 | y(k-7)^3 | -31.5 | 0.00253 | 37 | y(k-12)y(k-11)y(k-10) | -47.2 | 0.00105 |
| 13 | x1(k-11)x1(k-9)y(k-15) | 0.778 | 0.00198 | 38 | x1(k-14)x1(k-3)y(k-3) | -0.474 | 0.000956 |
| 14 | x1(k-7)x1(k-5)y(k-6) | -0.482 | 0.00188 | 39 | x1(k-14)x1(k-4)x1(k-3) | -0.0327 | 0.00113 |
| 15 | y(k-6)^2y(k-5) | -33.1 | 0.00166 | 40 | x1(k-14)y(k-7)y(k-3) | -6.79 | 0.000899 |
| 16 | x1(k-11)x1(k-4)y(k-13) | 0.649 | 0.00169 | 41 | y(k-10)y(k-7)^2 | 46.2 | 0.00126 |
| 17 | y(k-10)y(k-7) | -2.88 | 0.00154 | 42 | y(k-8)y(k-7)y(k-6) | 64.9 | 0.00104 |
| 18 | y(k-6)y(k-4) | 1.54 | 0.0016 | 43 | y(k-13)y(k-6)^2 | -25.4 | 0.00175 |
| 19 | x1(k-10)x1(k-9)y(k-7) | 0.355 | 0.00148 | 44 | y(k-12)y(k-1)^2 | 36.8 | 0.000951 |
| 20 | x1(k-5)y(k-3)y(k-2) | 2.9 | 0.00148 | 45 | y(k-14)y(k-3)y(k-2) | -67.2 | 0.000969 |
| 21 | x1(k-15)x1(k-14)x1(k-6) | -0.0373 | 0.00146 | 46 | y(k-12)y(k-5)y(k-4) | -42.1 | 0.000917 |
| 22 | x1(k-14)x1(k-5)y(k-8) | -0.516 | 0.00161 | 47 | y(k-2) | -0.104 | 0.000981 |
| 23 | x1(k-10)x1(k-3)x1(k-2) | 0.0327 | 0.00132 | 48 | y(k-9)y(k-2) | 1.38 | 0.00146 |
| 24 | x1(k-8)y(k-13)y(k-3) | 6.05 | 0.00161 | 49 | x1(k-15)x1(k-2)y(k-11) | 0.371 | 0.00101 |

| **Table A6: ADA-USD Sustainable** | | | | | | | |
| --- | --- | --- | --- | --- | --- | --- | --- |
| **Sr. #** | **Regressors** | **Parameters** | **ERR** | **Sr. #** | **Regressors** | **Parameters** | **ERR** |
| 0 | y(k-7) | 0.51 | 0.691 | 25 | x1(k-2)^2y(k-13) | 0.198 | 0.00153 |
| 1 | y(k-14) | 0.463 | 0.0556 | 26 | y(k-11)^2y(k-7) | -59 | 0.00154 |
| 2 | y(k-14)y(k-11)y(k-4) | -80 | 0.00798 | 27 | x1(k-12)y(k-7)y(k-5) | -4.07 | 0.00136 |
| 3 | y(k-14)y(k-10)^2 | -54.7 | 0.00462 | 28 | x1(k-12)^2 | -0.00721 | 0.00144 |
| 4 | y(k-14)^3 | -28.9 | 0.00443 | 29 | x1(k-15)y(k-15)y(k-8) | 5.26 | 0.00136 |
| 5 | y(k-15)y(k-14)y(k-7) | -37.4 | 0.00506 | 30 | y(k-12)y(k-7)y(k-3) | 64.5 | 0.00131 |
| 6 | y(k-1) | 0.12 | 0.00454 | 31 | y(k-11)y(k-9)y(k-1) | -66.1 | 0.00127 |
| 7 | y(k-12)y(k-5) | -2.12 | 0.00533 | 32 | y(k-2) | -0.18 | 0.00163 |
| 8 | y(k-7)y(k-2) | -2.49 | 0.00394 | 33 | x1(k-14)y(k-10)y(k-8) | 4.54 | 0.00131 |
| 9 | y(k-6)^2y(k-2) | -7.92 | 0.00283 | 34 | x1(k-11)x1(k-3)y(k-8) | 0.465 | 0.00124 |
| 10 | y(k-7)y(k-1) | -3.84 | 0.00371 | 35 | x1(k-3)y(k-9)^2 | 2.72 | 0.00128 |
| 11 | y(k-4)y(k-3)^2 | -39.9 | 0.00278 | 36 | y(k-9)y(k-2) | 2.2 | 0.00128 |
| 12 | y(k-7)^3 | -38.4 | 0.00253 | 37 | x1(k-11)x1(k-1)y(k-2) | -0.47 | 0.00128 |
| 13 | x1(k-15)x1(k-10)y(k-3) | 0.401 | 0.00192 | 38 | x1(k-6)x1(k-4)y(k-2) | 0.264 | 0.00137 |
| 14 | x1(k-15)x1(k-11)y(k-3) | 0.64 | 0.00204 | 39 | x1(k-15)y(k-15)y(k-10) | 5.31 | 0.00125 |
| 15 | x1(k-13)x1(k-7)y(k-3) | 0.544 | 0.00191 | 40 | y(k-7)y(k-2)^2 | -61.8 | 0.0013 |
| 16 | x1(k-7)x1(k-3)y(k-9) | -0.444 | 0.00195 | 41 | x1(k-9)y(k-14)y(k-11) | 5.31 | 0.00119 |
| 17 | y(k-15)^2y(k-14) | -62.4 | 0.0017 | 42 | x1(k-11)x1(k-7)y(k-7) | -0.359 | 0.00119 |
| 18 | x1(k-11)x1(k-2)y(k-13) | -0.639 | 0.00157 | 43 | y(k-12)y(k-1)^2 | 56.7 | 0.00108 |
| 19 | y(k-15)y(k-6)^2 | 45 | 0.00178 | 44 | y(k-15)y(k-12)y(k-10) | 60.8 | 0.00115 |
| 20 | y(k-6)y(k-2) | -2.65 | 0.00183 | 45 | y(k-13)y(k-9)y(k-5) | 72 | 0.00116 |
| 21 | y(k-8)y(k-7)y(k-6) | 80.1 | 0.00208 | 46 | x1(k-2)y(k-13)y(k-4) | 3.4 | 0.00122 |
| 22 | y(k-12)^2y(k-2) | -5.33 | 0.00187 | 47 | y(k-15)y(k-7)y(k-2) | 74.1 | 0.00107 |
| 23 | x1(k-15)x1(k-12) | 0.0177 | 0.00146 | 48 | x1(k-8)x1(k-6)^2 | -0.0172 | 0.00107 |
| 24 | x1(k-11)x1(k-8)y(k-8) | -0.415 | 0.00141 | 49 | x1(k-1)y(k-7)y(k-3) | -4.26 | 0.000912 |

| **Table A7: BITG-USD Sustainable** | | | | | | | |
| --- | --- | --- | --- | --- | --- | --- | --- |
| **Sr. #** | **Regressors** | **Parameters** | **ERR** | **Sr. #** |  |  |  |
| 0 | y(k-7) | 0.67 | 0.662 | 25 | x1(k-9)^2y(k-14) | -0.0262 | 0.00171 |
| 1 | y(k-14) | 0.225 | 0.0486 | 26 | x1(k-11)^2y(k-8) | -0.0307 | 0.00151 |
| 2 | y(k-11)y(k-7)y(k-4) | -123 | 0.00917 | 27 | x1(k-9)x1(k-7)x1(k-3) | 0.000759 | 0.00172 |
| 3 | y(k-10)^2y(k-7) | -46.4 | 0.00673 | 28 | x1(k-6)x1(k-5)y(k-7) | 0.0572 | 0.00161 |
| 4 | y(k-11)y(k-4)y(k-1) | 24.5 | 0.00516 | 29 | y(k-14)y(k-11)^2 | -39.7 | 0.00154 |
| 5 | y(k-2) | -0.237 | 0.00914 | 30 | x1(k-12)x1(k-4)x1(k-1) | 0.00184 | 0.00147 |
| 6 | y(k-4) | -0.125 | 0.00656 | 31 | x1(k-12)y(k-12)y(k-9) | 2.38 | 0.00149 |
| 7 | y(k-14)y(k-5) | 1.53 | 0.0053 | 32 | x1(k-13)x1(k-9)^2 | -0.00096 | 0.00152 |
| 8 | y(k-9)y(k-2) | 2.51 | 0.00717 | 33 | x1(k-14)x1(k-4)y(k-8) | 0.0504 | 0.00153 |
| 9 | y(k-6)^2y(k-1) | 36.7 | 0.00747 | 34 | x1(k-15)x1(k-8)y(k-14) | 0.0273 | 0.00145 |
| 10 | y(k-15)y(k-8)y(k-7) | -39.7 | 0.00429 | 35 | x1(k-5)^2y(k-2) | -0.0225 | 0.00139 |
| 11 | x1(k-11)x1(k-4)y(k-3) | -0.0662 | 0.00272 | 36 | x1(k-11)x1(k-10)x1(k-9) | -0.00104 | 0.00127 |
| 12 | y(k-7)^3 | -55.4 | 0.00215 | 37 | x1(k-11)y(k-10)^2 | -0.795 | 0.00123 |
| 13 | y(k-15)y(k-7)^2 | -53.7 | 0.0035 | 38 | x1(k-14)x1(k-11)x1(k-8) | 0.00082 | 0.00143 |
| 14 | y(k-7)y(k-2)^2 | -68.3 | 0.00246 | 39 | y(k-7)^2y(k-2) | -43.7 | 0.00138 |
| 15 | y(k-13)y(k-9)y(k-6) | 57.6 | 0.00228 | 40 | x1(k-3)x1(k-1)y(k-12) | 0.0242 | 0.00111 |
| 16 | x1(k-12)x1(k-11)y(k-10) | -0.0528 | 0.00191 | 41 | y(k-7)y(k-3)y(k-2) | -96.1 | 0.00111 |
| 17 | x1(k-15)x1(k-6)y(k-3) | 0.0433 | 0.00184 | 42 | y(k-1) | 0.1 | 0.00107 |
| 18 | x1(k-12)x1(k-1)y(k-1) | 0.0443 | 0.00166 | 43 | y(k-9)y(k-1) | 2.03 | 0.00141 |
| 19 | x1(k-14)x1(k-12)y(k-14) | 0.0422 | 0.00163 | 44 | y(k-7)y(k-2) | -2.51 | 0.0011 |
| 20 | x1(k-9)^2x1(k-8) | 0.000359 | 0.00179 | 45 | x1(k-4)y(k-8) | 0.0467 | 0.00107 |
| 21 | x1(k-5)x1(k-1)y(k-3) | 0.0617 | 0.00169 | 46 | x1(k-12)x1(k-11)x1(k-1) | -0.00103 | 0.00105 |
| 22 | y(k-12)^2 | -1.43 | 0.00153 | 47 | x1(k-10)y(k-9)y(k-6) | -2.24 | 0.0011 |
| 23 | x1(k-6)y(k-4) | 0.0819 | 0.00168 | 48 | x1(k-12)y(k-15)y(k-4) | 1.74 | 0.00111 |
| 24 | x1(k-6)y(k-11)y(k-5) | 1.69 | 0.00164 | 49 | y(k-12)y(k-6)y(k-4) | 33.3 | 0.000963 |

| **Table A8: MIOTA-USD Sustainable** | | | | | | | |
| --- | --- | --- | --- | --- | --- | --- | --- |
| **Sr. #** | **Regressors** | **Parameters** | **ERR** | **Sr. #** | **Regressors** | **Parameters** | **ERR** |
| 0 | y(k-7) | 0.367 | 0.691 | 25 | x1(k-2)y(k-14) | -0.12 | 0.0015 |
| 1 | y(k-14) | 0.541 | 0.0556 | 26 | y(k-10)y(k-6)y(k-4) | -54.8 | 0.00148 |
| 2 | y(k-14)y(k-11)y(k-4) | -104 | 0.00798 | 27 | x1(k-11)x1(k-6) | -0.0103 | 0.00136 |
| 3 | y(k-14)y(k-10)^2 | -59.4 | 0.00462 | 28 | x1(k-9)x1(k-8)y(k-8) | -0.271 | 0.00132 |
| 4 | y(k-14)^3 | -26.7 | 0.00443 | 29 | x1(k-13)y(k-15) | 0.127 | 0.00144 |
| 5 | y(k-15)y(k-14)y(k-7) | -51.5 | 0.00506 | 30 | x1(k-11)y(k-7)y(k-3) | 5.73 | 0.00127 |
| 6 | y(k-1) | 0.147 | 0.00454 | 31 | x1(k-5)^2y(k-14) | -0.16 | 0.00113 |
| 7 | y(k-12)y(k-5) | -0.449 | 0.00533 | 32 | x1(k-12)y(k-11)y(k-1) | 4.37 | 0.00108 |
| 8 | y(k-7)y(k-2) | -2.85 | 0.00394 | 33 | x1(k-10)x1(k-5)y(k-9) | 0.18 | 0.00113 |
| 9 | x1(k-4)y(k-7)y(k-4) | -8.07 | 0.00286 | 34 | x1(k-14)x1(k-3)y(k-1) | -0.227 | 0.00105 |
| 10 | y(k-4) | -0.133 | 0.00298 | 35 | x1(k-14)y(k-8)y(k-3) | 4.17 | 0.00143 |
| 11 | y(k-2) | -0.16 | 0.00316 | 36 | x1(k-14)y(k-7)y(k-3) | -4.19 | 0.00104 |
| 12 | y(k-2)y(k-1) | 2.84 | 0.00478 | 37 | x1(k-10)x1(k-7)x1(k-5) | -0.018 | 0.00106 |
| 13 | y(k-6)^2y(k-5) | -26.2 | 0.0025 | 38 | x1(k-9)x1(k-5)y(k-3) | -0.205 | 0.00107 |
| 14 | y(k-7)^3 | -23.8 | 0.00202 | 39 | x1(k-13)x1(k-11)y(k-15) | 0.26 | 0.00109 |
| 15 | x1(k-3)y(k-2)^2 | 1.87 | 0.00202 | 40 | x1(k-15)x1(k-13)x1(k-5) | -0.0218 | 0.001 |
| 16 | y(k-10)y(k-7)y(k-3) | -47.7 | 0.00172 | 41 | x1(k-15)x1(k-9)y(k-7) | 0.253 | 0.00108 |
| 17 | x1(k-9)y(k-10)y(k-4) | 3.28 | 0.00176 | 42 | x1(k-15)x1(k-2)y(k-2) | 0.208 | 0.00107 |
| 18 | x1(k-15)y(k-10)y(k-6) | -6.2 | 0.0016 | 43 | x1(k-7)x1(k-5)y(k-11) | 0.269 | 0.000924 |
| 19 | x1(k-15)x1(k-14)x1(k-8) | -0.0169 | 0.00171 | 44 | x1(k-15)^2y(k-12) | -0.117 | 0.000961 |
| 20 | x1(k-7)y(k-9)y(k-1) | 4.08 | 0.00159 | 45 | x1(k-8)x1(k-5)y(k-11) | 0.201 | 0.000894 |
| 21 | x1(k-15)y(k-8)y(k-4) | 6.95 | 0.00148 | 46 | y(k-12)^2 | -1.33 | 0.000901 |
| 22 | y(k-15)^2y(k-14) | -43 | 0.00154 | 47 | x1(k-6)y(k-11)y(k-2) | 2.24 | 0.000858 |
| 23 | x1(k-13)y(k-2) | -0.106 | 0.00146 | 48 | x1(k-13)y(k-7)y(k-4) | 3.91 | 0.000883 |
| 24 | x1(k-14)x1(k-13)x1(k-9) | 0.0205 | 0.00144 | 49 | y(k-15)y(k-12)y(k-4) | -57.3 | 0.000869 |

| **Table A9: XNO-USD Sustainable** | | | | | | | |
| --- | --- | --- | --- | --- | --- | --- | --- |
| **Sr. #** | **Regressors** | **Parameters** | **ERR** | **Sr. #** | **Regressors** | **Parameters** | **ERR** |
| 0 | y(k-7) | 0.509 | 0.683 | 25 | x1(k-7)x1(k-2) | -0.00758 | 0.00166 |
| 1 | y(k-14) | 0.311 | 0.0508 | 26 | y(k-13)y(k-9)y(k-5) | 57.5 | 0.00157 |
| 2 | y(k-11)y(k-7)y(k-4) | -138 | 0.00848 | 27 | x1(k-10)x1(k-5)y(k-1) | 0.182 | 0.00137 |
| 3 | y(k-10)^2y(k-7) | -40.9 | 0.00547 | 28 | x1(k-15)x1(k-11)^2 | 0.00361 | 0.00126 |
| 4 | x1(k-14)^2y(k-12) | -0.0839 | 0.00434 | 29 | y(k-6)^3 | -23.7 | 0.00116 |
| 5 | y(k-3) | -0.12 | 0.00444 | 30 | y(k-8)y(k-7)y(k-6) | 73.5 | 0.00128 |
| 6 | y(k-5)^2y(k-4) | -25.8 | 0.00452 | 31 | y(k-11)y(k-6)y(k-1) | 68.1 | 0.00131 |
| 7 | y(k-7)y(k-1) | -4.22 | 0.00467 | 32 | y(k-6)y(k-4) | 2.32 | 0.00125 |
| 8 | y(k-14)y(k-11)^2 | -54.9 | 0.00405 | 33 | y(k-14)y(k-7)y(k-6) | 48.2 | 0.00139 |
| 9 | y(k-2) | -0.179 | 0.00304 | 34 | y(k-12)y(k-9)y(k-2) | 46.8 | 0.00114 |
| 10 | y(k-11)y(k-4)y(k-1) | 96.8 | 0.00427 | 35 | x1(k-14)y(k-15)y(k-7) | -2.57 | 0.00108 |
| 11 | y(k-14)y(k-7)^2 | -75.2 | 0.00394 | 36 | x1(k-3)y(k-15)y(k-4) | -3.83 | 0.00113 |
| 12 | y(k-9)y(k-2) | 1.85 | 0.00447 | 37 | x1(k-9)y(k-8)y(k-3) | 2.28 | 0.00113 |
| 13 | y(k-12)^2 | -1.08 | 0.00435 | 38 | x1(k-6)x1(k-4)y(k-2) | 0.101 | 0.0011 |
| 14 | x1(k-8)x1(k-5)y(k-1) | 0.216 | 0.00222 | 39 | x1(k-13)x1(k-5)x1(k-2) | -0.0125 | 0.00109 |
| 15 | x1(k-2)y(k-6)y(k-2) | 4.31 | 0.00223 | 40 | y(k-7)y(k-3) | -2.15 | 0.00105 |
| 16 | y(k-11)y(k-7)y(k-6) | -71.4 | 0.00211 | 41 | y(k-10)^2y(k-6) | 48.5 | 0.000961 |
| 17 | y(k-13)y(k-10)y(k-6) | 49.2 | 0.00245 | 42 | x1(k-9)x1(k-4)x1(k-3) | 0.00696 | 0.000937 |
| 18 | x1(k-8)x1(k-5) | 0.00611 | 0.00195 | 43 | y(k-7)y(k-3)y(k-2) | -94.3 | 0.000928 |
| 19 | x1(k-1)y(k-7)y(k-3) | -3.18 | 0.00172 | 44 | y(k-7)y(k-2) | -3.44 | 0.00113 |
| 20 | y(k-14)y(k-3)^2 | -52 | 0.00243 | 45 | y(k-5) | -0.0648 | 0.00108 |
| 21 | x1(k-8)x1(k-3)y(k-3) | -0.204 | 0.00155 | 46 | x1(k-11)y(k-7)y(k-4) | 3.42 | 0.000987 |
| 22 | y(k-10)y(k-3) | 1.2 | 0.00162 | 47 | x1(k-13)y(k-15)y(k-4) | 3.95 | 0.00106 |
| 23 | y(k-15)y(k-14)y(k-9) | 63.2 | 0.00186 | 48 | y(k-7)y(k-2)^2 | -33.9 | 0.000988 |
| 24 | x1(k-15)x1(k-7)^2 | -0.00671 | 0.0016 | 49 | y(k-6)y(k-1)^2 | -25.7 | 0.000861 |

| **Table A10: POWR-USD Sustainable** | | | | | | | |
| --- | --- | --- | --- | --- | --- | --- | --- |
| **Sr. #** | **Regressors** | **Parameters** | **ERR** | **Sr. #** | **Regressors** | **Parameters** | **ERR** |
| 0 | y(k-7) | 0.368 | 0.691 | 25 | x1(k-14)y(k-13)y(k-12) | -1.22 | 0.00111 |
| 1 | y(k-14) | 0.554 | 0.0556 | 26 | y(k-14)y(k-12) | 3.08 | 0.00108 |
| 2 | y(k-14)y(k-11)y(k-4) | -65.1 | 0.00798 | 27 | y(k-6)y(k-4) | 1.06 | 0.00125 |
| 3 | y(k-14)y(k-10)^2 | -58 | 0.00462 | 28 | y(k-10)y(k-1) | -1.88 | 0.00111 |
| 4 | y(k-14)^3 | -13.6 | 0.00443 | 29 | y(k-14)^2y(k-7) | -68.5 | 0.00117 |
| 5 | y(k-15)y(k-14)y(k-7) | -7.7 | 0.00506 | 30 | x1(k-4)y(k-7)y(k-2) | -0.88 | 0.00122 |
| 6 | y(k-1) | 0.141 | 0.00454 | 31 | y(k-14)y(k-11)^2 | -46.2 | 0.000997 |
| 7 | y(k-12)y(k-5) | -1.11 | 0.00533 | 32 | x1(k-7)^2y(k-10) | 0.0116 | 0.000952 |
| 8 | y(k-7)y(k-2) | -2.84 | 0.00394 | 33 | x1(k-9)y(k-11)y(k-1) | -1.54 | 0.000912 |
| 9 | x1(k-13)x1(k-2)y(k-2) | -0.0347 | 0.00298 | 34 | x1(k-9)x1(k-2)y(k-1) | -0.0141 | 0.000989 |
| 10 | y(k-12)y(k-5)y(k-4) | -39.1 | 0.00269 | 35 | y(k-13)y(k-12)y(k-9) | 85.1 | 0.000932 |
| 11 | y(k-14)y(k-9)y(k-1) | 70.3 | 0.0028 | 36 | y(k-13)y(k-12)y(k-8) | -72 | 0.00109 |
| 12 | y(k-6)^2y(k-2) | -43.4 | 0.00285 | 37 | y(k-9)y(k-7)y(k-6) | 66 | 0.00115 |
| 13 | y(k-7)^3 | -22 | 0.00177 | 38 | y(k-8)y(k-6)^2 | 22.4 | 0.000994 |
| 14 | y(k-7)y(k-1) | -4.78 | 0.0022 | 39 | y(k-10)^2y(k-6) | 43.3 | 0.000901 |
| 15 | x1(k-3)y(k-7)y(k-3) | -3.72 | 0.0019 | 40 | x1(k-5)y(k-10)y(k-6) | 2.28 | 0.000928 |
| 16 | x1(k-10)x1(k-3)y(k-15) | 0.0249 | 0.00179 | 41 | x1(k-5)y(k-7)y(k-3) | -2.26 | 0.00102 |
| 17 | y(k-6)y(k-2) | -2.66 | 0.00162 | 42 | y(k-7)y(k-5)y(k-3) | 55.9 | 0.000975 |
| 18 | y(k-12)^2y(k-2) | -56.9 | 0.00198 | 43 | x1(k-13)y(k-7)y(k-2) | 1.99 | 0.001 |
| 19 | y(k-13)y(k-7)y(k-5) | -32.3 | 0.00158 | 44 | x1(k-4)y(k-7)y(k-3) | -1.52 | 0.000846 |
| 20 | y(k-14)y(k-7) | 4.34 | 0.00152 | 45 | x1(k-15)x1(k-13) | 0.000575 | 0.000804 |
| 21 | y(k-14)y(k-1)^2 | -65.8 | 0.00185 | 46 | x1(k-13)x1(k-5)y(k-13) | 0.0322 | 0.000864 |
| 22 | y(k-6)^2y(k-5) | -29.6 | 0.00164 | 47 | x1(k-13)y(k-14)y(k-7) | 0.92 | 0.00085 |
| 23 | x1(k-8)y(k-12)^2 | 0.826 | 0.00128 | 48 | y(k-14)y(k-11)y(k-2) | 49.4 | 0.000842 |
| 24 | x1(k-3)y(k-5)y(k-1) | 1.96 | 0.00119 | 49 | x1(k-12)x1(k-7) | -0.00054 | 0.000795 |
